# Genus-level evolutionary relationships of FAR proteins reflect the diversity of lifestyles of free-living and parasitic nematodes

**DOI:** 10.1186/s12915-021-01111-3

**Published:** 2021-08-30

**Authors:** Dongjuan Yuan, Song Li, Ziyu Shang, Muchun Wan, Yu Lin, Yanhua Zhang, Yaoyu Feng, Lian Xu, Lihua Xiao

**Affiliations:** 1 Center for Emerging and Zoonotic Diseases, College of Veterinary Medicine, South China Agricultural University, Guangzhou, 510642 China; 2 Guangdong Laboratory for Lingnan Modern Agriculture, Guangzhou, 510642 China; 3 Department of Parasitology, Zhongshan School of Medicine, Sun Yat-sen University, Guangzhou, 510080 China; 4 Key Laboratory of Neuroregeneration of Jiangsu and Ministry of Education, Co-innovation Center of Neuroregeneration, Nantong University, Nantong, 226019 China

**Keywords:** Fatty acid and retinol-binding protein, Evolution, Expansion, Nematoda, Transcriptome

## Abstract

**Background:**

Nematodes are a widespread and diverse group comprising free-living and parasitic species, some of which have major detrimental effects on crops, animals, and human health. Genomic comparisons of nematodes may help reveal the genetic bases for the evolution of parasitic lifestyles. Fatty acid and retinol-binding proteins (FARs) are thought to be unique to nematodes and play essential roles in their development, reproduction, infection, and possibly parasitism through promoting the uptake, transport, and distribution of lipid and retinol. However, the evolution of FAR family proteins across the phylum Nematoda remains elusive.

**Results:**

We report here the evolutionary relationship of the FAR gene family across nematodes. No FAR was found in Trichocephalida species and *Romanomermis culicivorax* from Clade I, and FAR could be found in species from Clades III, IV, and V. FAR proteins are conserved in Clade III species and separated into three clusters. Tandem duplications and high divergence events lead to variable richness and low homology of FARs in *Steinernema* of Clade IVa, *Strongyloides* of Clade IVb, and intestinal parasitic nematodes from Clades Vc and Ve. Moreover, different richness and sequence variations of FARs in pine wood, root-knot, stem, and cyst nematodes might be determined by reproduction mode or parasitism. However, murine lungworm *Angiostrongylus* and bovine lungworm *Dictyocaulus viviparus* from Clade Vd have only 3–4 orthologs of FAR. RNA-seq data showed that *far* genes, especially *far*-1 and *far*-2, were highly expressed in most nematodes. *Angiostrongylus cantonensis* FAR-1 and FAR-3 have low sequence homology and distinct ligand-binding properties, leading to differences in the cavity volume of proteins. These data indicate that FAR proteins diverged early and experienced low selective pressure to form genus-level diversity. The *far* genes are present in endophyte or root-colonized bacteria of *Streptomyces*, *Kitasatospora* sp., *Bacillus subtilis*, and *Lysobacter*, suggesting that bacterial *far* genes might be derived from plant-parasitic nematodes by horizontal gene transfer.

**Conclusions:**

Data from these comparative analyses have provided insights into genus-level diversity of FAR proteins in the phylum Nematoda. FAR diversification provides a glimpse into the complicated evolution history across free-living and parasitic nematodes.

**Supplementary Information:**

The online version contains supplementary material available at 10.1186/s12915-021-01111-3.

## Background

Parasitic nematodes infect animals and plants as well as human beings, causing detrimental impacts on economic crops, farm animals, and human health. Comparative genomics studies indicated that the fewer orthologs were found in the lipid biosynthesis and metabolism of parasitic nematodes than in free-living *C. elegans* [1–3]. Lipids are hydrophobic components, including fatty acids, phospholipids, cholesterol, steroids, and fat-soluble vitamins. Lipids play diverse roles in regulating physiological and pathological functions of organisms. Fatty acids are important components in the synthesis and construction of epidermis and influence embryo development [4–6]. Polyunsaturated fatty acids (PUFAs) and their metabolites are signaling molecules and participate in regulating signal transduction and post-translational modifications to promote the development, reproduction, and lifespan of worms and are even involved in pathogenic processes following nematode infection of a host [7–12]. Retinol or retinoic acid and fat-soluble vitamins direct programmed spermatogonial and meiotic differentiation that are essential for the generation of functional spermatozoa [13, 14]. Furthermore, retinoic acid also affects a wide variety of biological membranes and plays an important role in regulating signaling pathways and tissue differentiation, tissue repairs, and the IgA and Th2 cytokine levels [15]. Due to the fewer orthologs in fatty acid biosynthesis and metabolism pathways compared to free-living *C. elegans* [1, 2], parasitic nematodes might rely on lipid binding and transport proteins to absorb, transport, and phagocytose various lipid or metabolic molecules from their hosts.

Fatty acid and retinol-binding protein (FAR), first discovered in *Onchocerca volvulus* [16], is a secretory protein. The mRNA for its expression was localized in the hypodermis below the cuticle in in situ hybridization studies of plant-parasitic nematodes [17, 18]. FAR is widely known as a unique protein in nematodes, engaging in promoting the uptake, transport, and distribution of lipid and retinol. Comparative genomics studies showed the presence of FAR expansions in some *Steinernema* spp. and strongylids [19, 20]. Functional studies through gene silencing indicated that *far-1* could regulate the development and reproduction of nematodes [17, 21–23]. A few reports showed that FAR could affect the infection and pathogenicity of nematodes in plants [18, 24, 25]. However, the evolution of FAR across the phylum Nematoda remains elusive.

Growing genomic and transcriptomic data are now available for many nematodes, including free-living nematodes of *C. elegans*, *Rhabditophanes* sp., *Pristinochus*, and *Diploscapter coronatus*; pine wood, stem, root-knot, and cyst nematodes of plants; whipworm, *Ascaris*, *Onchocerca*, lymphatic filaria, hookworm; other nematodes of veterinary importance; and insect-parasitic nematodes *Romanomermis culicivorax* and *Steinernema* spp. In the present study, we analyzed gene number, structure, origin, evolution of FAR in 58 nematodes, and transcription pattern of the *far* gene across developmental stages of several nematodes. We detected genes encoding FAR proteins in genomes of most nematodes as well as trematode *Schistosome mansoni*, cestode *Echinococcus multilocularis*, free-living *Schmidtea mediterranea*, and bacteria. We further assessed ligand-binding properties and structure of FAR protein from *Angiostrongylus cantonensis*, the rat lung worm.

## Results

### Loss, duplication, and genus-level expansion of *far* genes in nematodes

**Fig. 1:**
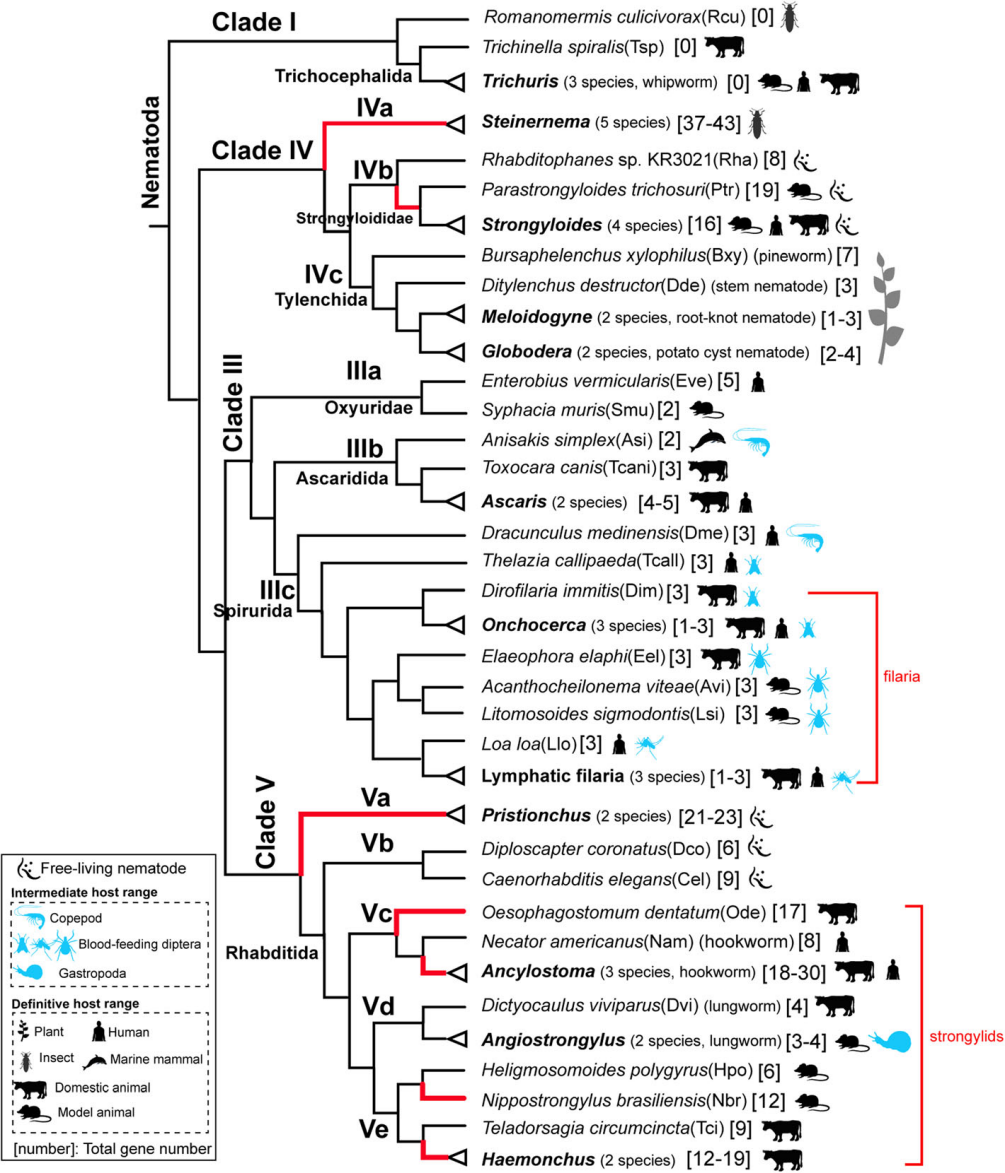
Comparison of Gene Numbers of *far* in 58 nematodes. The range of definitive hosts and intermediate hosts of the nematodes is shown. Taxonomic classification of nematodes was retrieved from the Taxonomy database. The topology of Nematoda phylogeny was inferred as described [26]. Genus names are shown in bold italics and the species numbers are shown in parentheses. The number of *far* genes is shown in square brackets. Red branch represents the species with expanded *far* genes. *Trichuris* species include *T. muris* (Tmu), *T. suis* (Tsu), and *T. trichiura* (Ttr); *Steinernema* species include *S. carpocapsae* (Sca), *S. feltiae* (Sfe), *S. glaseri* (Sgl), *S. monticolum* (Smo), and *S. scapterisci* (Ssc); *Strongyloides* species include *S. papillosus* (Spa), *S. ratti* (Sra), *S. stercoralis* (Sst), and *S. venezuelensis* (Sve); *Meloidogyne* species include *M. hapla* (Mha) and *M. incognita* (Min); *Globodera* species includes *G. pallida* (Gpa) and *G. rostochiensis* (Gro); *Ascari*s species include *A. lumbricoides* (Alu) and *A. suum* (Asu); *Onchocerca* species include *O. flexuosa* (Ofl), *O. ochengi* (Ooc), *O. volvulus* (Ovo); Lymphatic filaria include *Wuchereria bancrofti* (Wba), *Brugia malayi* (Bma), and *Brugia pahangi* (Bpa); *Pristionchus* species include *P. exspectatus* (Pex) and *P. pacificus* (Ppa); *Ancylostoma* include *A. caninum* (Aca), *A. ceylanicum* (Ace), and *A. duodenale* (Adu); *Angiostrongylus* include *A. costaricensis* (Aco) and *A. cantonensis* (Acant); *Haemonchus* include *H. contortus* (Hco) and *H. placei* (Hpl)

We found 586 FAR proteins from 58 nematode species by searching for the Gp-FAR-1 domain (pfam05823, Additional file 2: Table S1). The FAR domain was not found in 5 species from Clade I, but present in Clades III, IV, and V. The median number of *far* genes in 53 species from Clades III, IV, and V was 5. The number of the *far* genes varied at the genus level (Fig. 1 and Additional file 3: Table S2). The number of the *far* genes ranged from 1 to 5 in Clade III, and being 3 in most species in Clade IIIc. In Clade IV, the number of the *far* genes in plant-parasitic nematodes (1-7, in Clade IVc) was significantly lower than in entomopathogenic nematode *Steinernema* (37-43, in Clade IVa) and parthenogenetic nematode *Strongyloides* (16, in Clade IVb) (Fig. 1 and Additional file 1: Fig. S1). Variations in numbers of *far* genes in Clade V was detected not only among free-living nematodes *C. elegans* (9, in Clade Vb), *D. coronatus* (6, in Clade Vb), and *Pristinochus* (21-23, in Clade Va), but also among parasitic nematodes of *Angiostrongylus* (3-4, in Clade Vd), *Dictyocaulus viviparus* (4, in Clade Vd), and expanded *Ancylostoma* (18-30, in Clade Vc), *Nippostrongylus brasiliensis* (12, in Clade Ve), and *Haemonchus* (12-19, in Clade Ve) (Fig. 1 and Additional file 1: Fig. S1). Thus, the lack or expansion of gene was responsible for the extensive variations in the number of *far* genes in nematodes of different genera.

### Low sequence identity and high diversity of FAR proteins at the genus level within phylum Nematoda

The sequence identity of FAR proteins was compared among members within the phylum Nematoda. The average sequence identity between the FAR domain in 586 nematode proteins and the Gp-FAR-1 domain was 23.9% (Additional file 2: Table S1). The average sequence identities among 55 FAR domains from Clade III, 310 from Clade IV, and 221 from Clade V were 29.1%, 17.1%, and 21.2%, respectively (Additional file 1: Fig. S2, S3, S4, and Additional file 2: Table S1). We named orthologs in species with serial numbering according to the sequence identity to Gp-FAR-1 and known nematode FAR-1 s (Additional file 2: Table S1). We used OrthoMCL to infer the orthologous relationship of the 586 protein sequences with FAR domains among free-living and parasitic nematodes. The results obtained showed sequence divergence of FAR proteins across nematodes, categorizing them into 18 groups. A ML tree of the FAR domains across nematodes was further constructed. Expansions in FAR domains in species from three clades had led to the formation of several monophyletic groups (Additional file 1: Fig. S5), which make the phylogenetic tree complicated. It illustrates variances in gene numbers and low sequence homology among FAR proteins. These features reflect the complex genetic relationship of FAR among nematodes. Thus, we constructed phylogenetic trees of FAR for some species from these nematode clades.

### Three isoforms of FAR in the ancestor of Clade III

**Fig. 2:**
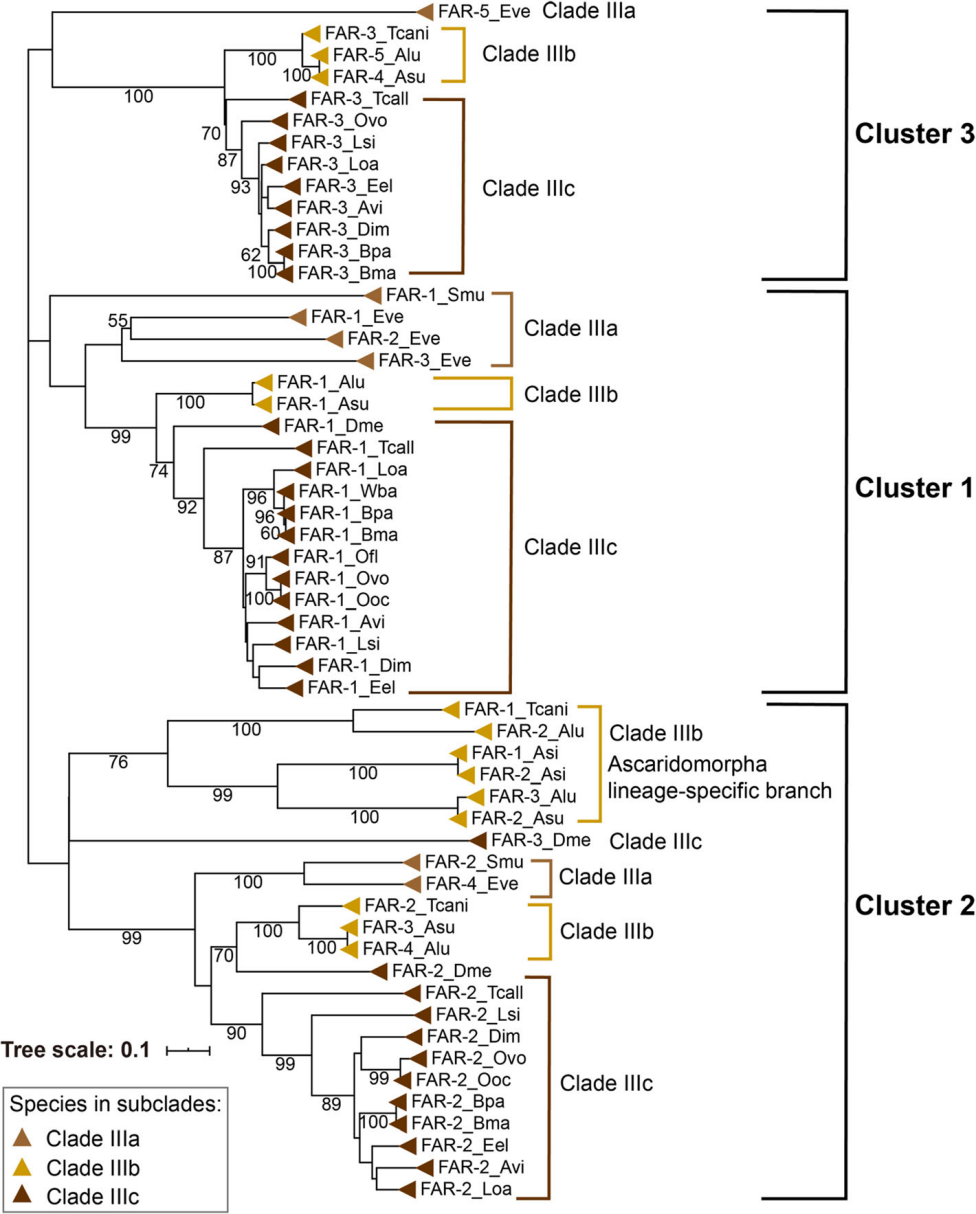
Protein phylogenetic tree of FARs among nematodes from Clade III. Bootstrap values are shown in the nodes. The scale bar represents the number of amino acid substitutions per site. The triangles with different colors represent FAR proteins in species from Clades IIIa, IIIb, and IIIc. Gene name includes the abbreviation of species name, as depicted in Fig. 1

FAR proteins grouped into one cluster in phylogenetic tree are expected to include members of nematode species of the same subclade within Clade III. Indeed, 55 ortholog sequences of FAR domain from Clade III formed three clusters (Fig. 2). Most FAR-1 proteins were grouped into cluster 1 and showed sequence identity of 49.4%. In cluster 2, most FAR-2 proteins from Clade III were grouped together, but some FARs from Clade IIIb were clustered into Ascaridomorpha lineage-specific branch. Cluster 3 contained most FAR-3 from Clade IIIc, three FARs of Ascaridida in Clade IIIb, and FAR-5 of *Enterobius vermicularis* in Clade IIIa. *Far* genes from Clade IIIa and IIIc shared similar numbers and length of introns, particularly in Spirurida (Additional file 1: Fig. S6). *Far* genes in Ascaridida had more and longer introns than others in Clade III, suggesting that *far* genes in Ascaridida have been separated from other species in Clade III at their ancestors. Two FARs of marine nematode *A. simplex* in Clade IIIb clustered together into the Ascaridomorpha lineage-specific branch and shared intron structure like *far* genes in Clade IIIa and IIIc, suggesting that *far* genes in *A. simplex* might have experienced losses in the evolutionary process. Thus, the *far* genes from Clade III might have separated into three clusters at their ancestor of Oxyuridae, Ascaridida, and Spirurida. Moreover, the ancestor of Ascaridida has experienced gene duplication of *far* genes.

### Extensive expansion and divergence of FAR proteins in Clade IV

**Fig. 3:**
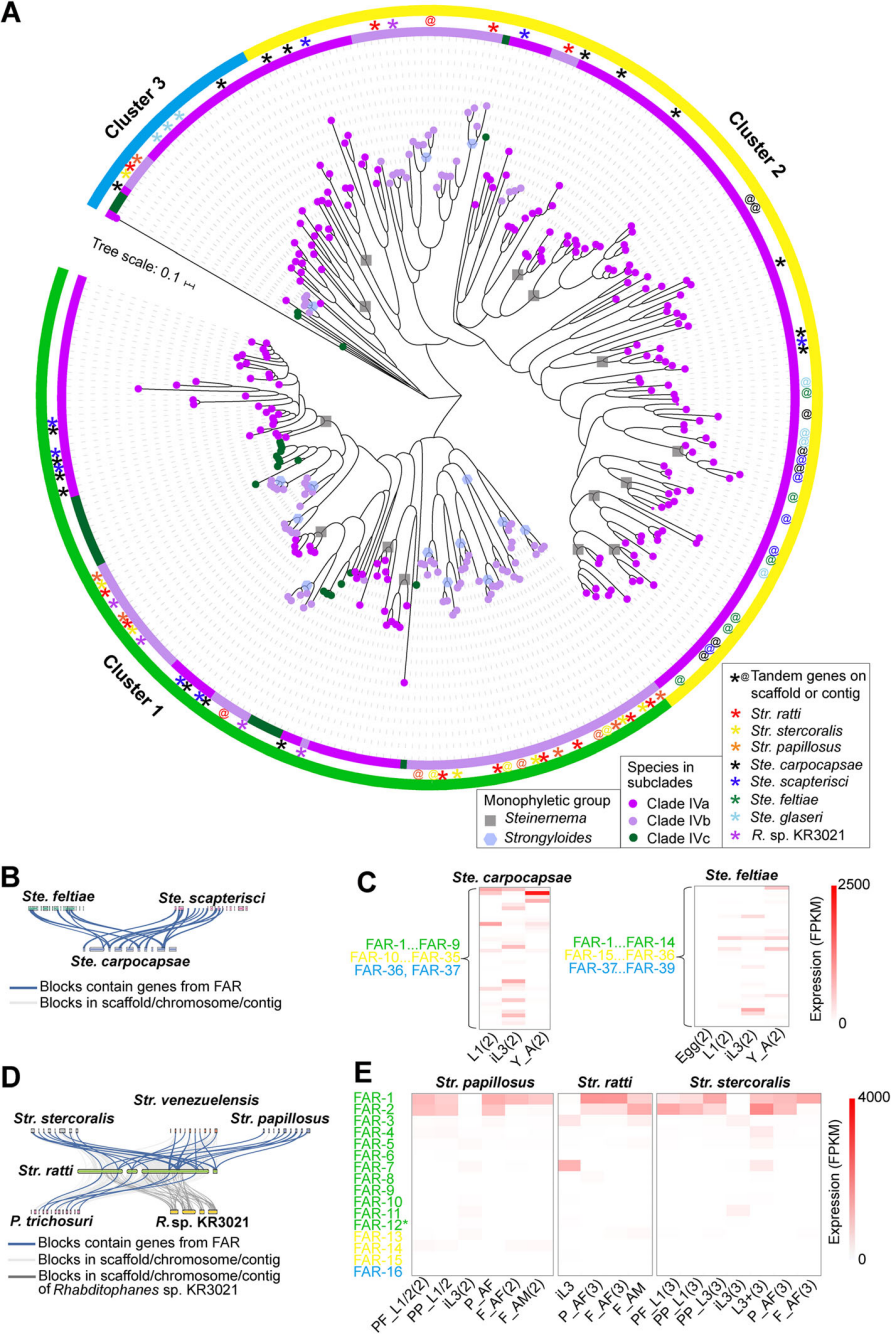
Evolution of FARs among nematodes from Clade IV. **A** Maximum-Likelihood tree of 310 FAR proteins from nematodes in Clade IV. The scale bar represents the number of amino acid substitutions per site. The circles with different colors on the branches represent FAR protein from Clades IVa, IVb, and IVc. The color of the inner ring is corresponding to the color of solid circle on the branch. The green, yellow, and blue blocks on the outer ring represent clusters 1, 2, and 3, respectively. The dark gray box and the blue purple hexagon represent the monophyletic groups of *Steinernema* and *Strongyloides*, respectively. * and @ indicate tandem replication on scaffolds or contigs, respectively. **B**, **D** Numbers of 1:1 far orthologs that are syntenic in *Ste. carpocapsae* and other *Steinernema* species. Syntenic blocks in scaffolds or contigs containing far genes in species from Clades IVa and IVb. The blue line represents far orthologs with collinearity on the genome of these species, and the gray line represents far orthologs without collinearity on the genome of these species. **C**, **E** Expression pattern of far genes across developmental stages in two *Steinernema* species and four *Strongyloides* species with available RNA-seq data. Detailed expression values are shown in Additional file 4: Table S3. * means Str. ratti FAR-12 was grouped into cluster 2 but not cluster 1. iL3: infective third-stage larvae, Y_A:young adult, AF:adult female, Y_AF:young adult female, P_AF:parasitized adult female, F_AF:free-living adult female

FAR proteins in genera *Steinernema* of Clade IVa and *Strongyloides* of Clade IVb have significantly expanded to more than 37 and 16, respectively, while those in plant-parasitic nematodes of Clade IVc ranged from 1 to 7. The ML tree of FARs in Clade IV formed three clusters. The expanded FARs are seen as several *Steinernema*-specific and *Strongyloides*-specific groups in different clusters. In cluster 1, we observed 4 monophyletic groups from five *Steinernema* species and 11 monophyletic groups from four *Strongyloides* species (Fig. 3A and Additional file 2: Fig. S7). In cluster 2, we observed 9 monophyletic groups from five *Steinernema* species and 2 monophyletic groups from four *Strongyloides* species. Some FARs from *Steinernema* species had independent expansion. In cluster 3, FARs from five *Steinernema* species and four *Strongyloides* species formed one monophyletic group separately; moreover, FARs from *Ste. monticolum* and *Ste. glaseri* appeared to have gone through expansions. Thus, FARs in five *Steinernema* species and four *Strongyloides* species had experienced expansions and formed at least 14 monophyletic groups. In addition, FARs from plant-parasitic nematode *Meloidogyne*, *Globodera*, and *Ditylenchus destructor* were only grouped into cluster 1, while FARs from *Bursaphelenchus* had lineage-specific expansions and were grouped into three clusters.

#### FARs arose independently in entomopathogenic lineage of Steinernema

*Steinernema* spp. are entomopathogenic nematodes, which can kill an insect host within 24–48 h [27, 28]. *Steinernema* species have more than 38 FARs with significant sequence divergence. The FAR gene family represents the dramatic case of genus-wide expansion in *Steinernema* genomes [20]. Some expanded *far* genes were tandem ones that had higher homology and closer phylogenetic relationship (Fig. 3A and Additional file 1: Fig. S7 and S8). We also examined the synteny of *far* gene of *Steinernema* in chromosomes or scaffolds (gene number in a scaffold of more than six genes were considered; information on scaffolds/contigs encoding *far* gene is listed in Additional file 2: Table S1). The gene order in the syntenic block containing the *far* gene was highly conserved among *Ste. carpocapsae*, *Ste. feltiae*, and *Ste. scapterisci* (Fig. 3B). The expression of *far* genes across developmental stages showed two divergent expression patterns. Some *far* genes had high expression in infective L3 stage; others had high expression in L1 and young adult stages, while *far* genes in *Ste. feltiae* showed low expression in egg stage (Fig. 3C), according to the data of Dillman AR et al. We found no *far* gene in insect-parasitic *R. culicivorax* from Clade I and three *far* genes in entomopathogenic *Heterorhabditis bacteriophora* (PRJNA13977) [2], which may illustrate independent evolution of *far* genes in insect-parasitic *Steinernema* lineages.

#### Tandem FAR-1 and FAR-2 are possibly related with *Strongyloides* development

Clade IVb contains the free-living *Rhabditophanes* sp. KR3021 from the Alloionematidae family and parasitic *Parastrongyloides trichosuri* and *Strongyloides* from the Strongyloididae family. The 16 *far* genes in *Strongyloides* and 19 in *P. trichosuri* as a result of gene expansion formed three clusters (Fig. 3A and Additional file 1: Fig. S7). Some of the expanded *far* genes from *Strongyloides* in cluster 1 are in tandem and have high sequence homology and close phylogeny relationship (Fig. 3A and Additional file 1: Fig. S7 and S8). We also assessed gene synteny in chromosomes or scaffolds containing *far* among *Strongyloides*, *P. trichosuri*, and *Rhabditophanes* sp. KR3021 (Additional file 2: Table S1). The results showed that gene order in the syntenic blocks is highly conserved between *Strongyloides* and *P. trichosuri*, but not between *S. ratti* and *Rhabditophanes* sp. KR3021 (Fig. 3D). *Far* had two exons in Strongyloididae and three exons in free-living *Rhabditophanes* sp. KR3021 (Additional file 3: Table S2), suggesting that *far* gene experienced intron losses in the last common ancestor of Strongyloididae. These data indicate that *far* genes in free-living *Rhabditophanes* and parasitic Strongyloididae had diverged early.

RNA-seq data from *Strongyloides* spp. in public database [29–31] enables us to investigate the potential roles of genes in nematode biology. Analysis of transcriptomic data from three *Strongyloides* species showed that the *far-1* and *far-2* genes had coordinately higher expression than other *far* genes. Moreover, low expression of *far* was observed in iL3 compared with other developmental stages in four *Strongyloides* species (Fig. 3E and Additional file 4: Table S3). *Strongyloides* spp. are female-only in parasitic lifestyle and dioecious in free-living lifestyle. The *far* genes in free-living or parasitic females had similar gene expression level. Analysis of somatic proteomes of free-living and parasitic females of *Str. ratti* showed that FAR-1 (original gene id: SRAE_2000289100) and FAR-2 (original gene id: SRAE_2000289500) had high expression in free-living and parasitic stages [30]. In addition, FAR-1 and FAR-2 could be detected in excretory-secretory (ES) proteome of *Str. ratti* [30, 32], reflecting its importance in the host-nematode interaction. Thus, considering the high expression level of *far-1* and *far-2* genes in free-living and parasitic females, and its presence in ES, we propose that at least FAR-1 and FAR-2 in *Strongyloides* might be important in its development and parasitism.

#### FAR represents the evolutionary dynamic of plant-parasitic nematodes

Orthologs of FAR have diverse phylogeny relationship among pine wood, root-knot, stem, and cyst nematodes (Fig. 3A and Additional file 1: Fig. S7). In cluster 1, FARs from root-knot, pine wood, stem, and cyst nematodes were clustered together, and other FARs from pine wood, stem, and cyst nematodes were grouped into another group, while FARs from *B. xylophilus* had lineage-specific expansion and were clustered in three clusters (Fig. 3A and Additional file 1: Fig. S7). To elucidate the evolutionary relationship of FAR in plant-parasitic nematodes, we conducted comparative analyses of genomes of seven divergent plant-parasitic nematodes: the root-knot nematodes *M. graminicola*, *M. floridensis*, *M. arenaria*, *M. javanica*, and *M. enterolobii*; the cyst nematode *Heterodera glycines*; and the pine wood nematode *B. okinawaensis*.

**Fig. 4:**
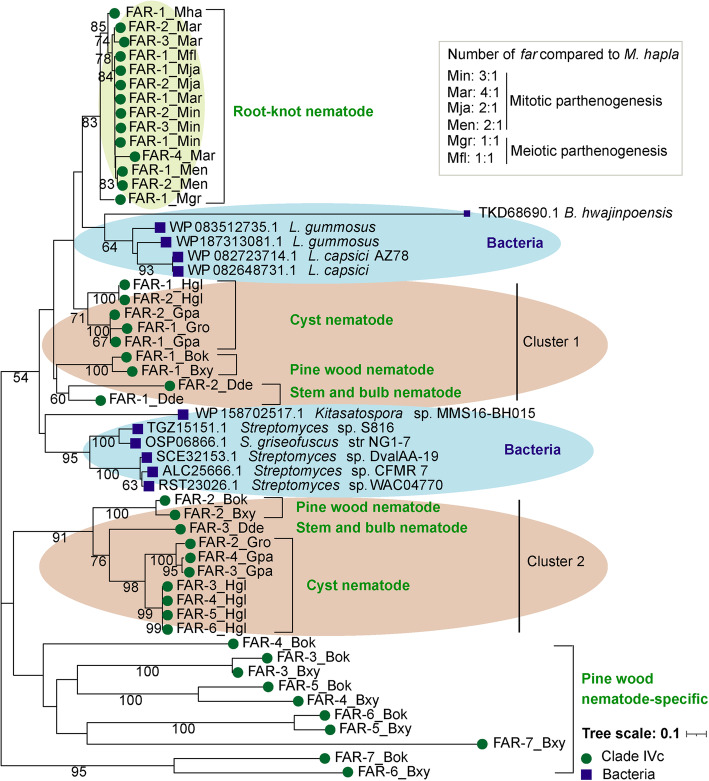
Phylogenetic Tree of FAR Proteins from Plant-parasitic Nematodes and Bacteria. Ratio (1:1, 2:1, and ≥ 3:1) of *far* gene number shared between a *Meloidogyne* species and the meiotic parthenogenesis of *M. hapla* (act as the baseline) in root-knot nematodes were calculated. Bootstrap values are shown in the nodes. The scale bar represents the number of amino acid substitutions per site. The green circle represents FAR protein from Clade IVc, and the deep-blue rectangle represents FAR protein from bacteria. The protein name includes abbreviated species name, as depicted in Fig. 1

In phylogenetic analyses of the sequences, FARs from root-knot nematodes clustered together (Figs. 3A and 4 and Additional file 1: Fig. S7). One *far* gene is present in the genomes of *M. hapla*, *M. graminicola*, and *M. floridensis*, but two to four FARs were encoded in the genomes of *M. incognita*, *M. arenaria*, *M. javanica*, and *M. enterolobii*. To determine whether the latter might have originated from gene duplication, we analyzed the reproduction mode and other features. The reproduction mode in root-knot nematode is complex and different from that of other plant-parasitic nematodes. Some of root-knot nematodes have facultative meiotic parthenogenesis (*M. hapla*, *M. graminicola*, and *M. floridensis*), while others are obligatory mitotic parthenogenesis (*M. incognita*, *M. arenaria*, *M. javanica*, and *M. enterolobii*), which lead to the aneuploid and polyploid genomes [33]. The ratio of *far* gene number in mitotic parthenogenetic species to meiotic parthenogenetic *M. hapla* is approximately 2:1 or more than 3:1 (Fig. 4). Our previous study showed that the proportion of the duplicated BUSCOs (13.1–36.7%) in four mitotic parthenogenetic species was higher than in three meiotic parthenogenetic species (0.4–3.0%). The ratio of these BUSCOs number in root-knot nematodes to *M. hapla* with 2:1 or 3:1 reached to 26–42% in four mitotic parthenogenetic species, particularly in *M. arenaria*, while was less than 5% in two meiotic parthenogenetic species [34]*.* Thus, the multi-copy nature of *far* gene in mitotic parthenogenetic species was likely due to their genomic characteristics. The analysis of RNA-seq data indicated that *far-1* and *far-2* genes had relatively high expression across developmental stages of *M. incognita* (Additional file 1: Fig. S9B and Additional file 4: Table S3).

Results of phylogenetic analysis showed that FARs in pine wood, stem, and cyst nematodes were grouped into two clusters, with duplicated FAR of *Bursaphelenchus* in a separate branch (Fig. 4). The *far* genes in *Globodera rostochiensis* and *Globodera pallida* shared similar intron structure, while differed from the orthologs in *H. glycines* (Additional file 1: Fig. S9A). Species in *Globodera* and *Heterodera* had been diverged over 30 million years ago, and the *far* genes in *G. pallida* and *H. glycines* might have experienced independent duplications in the evolutionary process. The genome of pine wood nematode *B. okinawaensis* had seven *far* genes, which is consistent with those in *B. xylophilus.* Phylogenetic analysis indicated that the lineage-specific *far* genes occurred and duplicated in their last common ancestor (Figs. 3A and 4 and Additional file 1: Fig. S7). RNA-seq data indicated that *far-1* and *far-2* in *B. xylophilus* had relatively high expression across developmental stages, and the expression level of *far-1* was higher than *far-2* (Additional file 1: Fig. S9B and Additional file 4: Table S3). Lineage-specific expansions and high expression of *far-1* and *far-2* in infective or parasitic stages may be advantageous to the parasitism of pinewood nematode.

#### Comparison of FAR from plant-parasitic nematode and bacteria

A comprehensive homology searching of whole genome sequence data showed the presence of FAR domains in bacteria *Streptomyces*, *Kitasatospora* sp., *Bacillus subtilis*, and *Lysobacter.* Results of sequence identity and phylogenetic analyses indicated that bacterial FAR domains had higher sequence identity to those in plant-parasitic nematodes than in other nematodes, especially to FAR-1 in plant-parasitic nematodes (Additional file 1: Fig. S10A). Bacterial *far* genes, however, have no intron (Additional file 1: Fig. S10B). We observed genome collinearity in the coding sequence (CDS) region of FAR domains between plant-parasitic nematodes and these bacteria. The gene spacing and orientation of FAR domains were conserved between them, which was not the case between the bacteria and other nematodes (Additional file 1: Fig. S11 and Fig. S12). There were extensive differences between *far* genes and other genes in GC content, gene combination, and codon usage bias. The GC content in bacterial genomes (66.5-72.1% in *Lysobacter*, *Kitasatospora* sp., and *Streptomyces*) was significantly higher than in plant-parasitic nematode genomes (23.5% - 40.4%) (Additional file 5: Table S4). The average GC content between all CDS and FAR domains were 48.3% and 47.6% (*P* = 0.38) in plant-parasitic nematodes, 44.5% and 42.4% (*P* = 0.26) in other nematodes from different clades, and 72.3% and 59.2% (*P* = 0.0000006) in bacterial CDS and FAR domains, respectively (Additional file 5: Table S4). Thus, bacterial *far* genes had GC contents compared different significantly from the whole genomes. Because of the difference in GC content, the codon usage frequency of bacterial *far* genes was compared with that of other genes in the genomes of bacteria. The results obtained showed that the ratio of the five codon indices (CAI, Fop, Nc, GC3s, and GC) of *far* genes to the whole genome was about 1 (Additional file 5: Table S5). Therefore, the codon index of bacterial *far* genes was similar to that of the whole genome of bacteria. *Streptomyces*, *Kitasatospora* sp., and *Bacillus subtilis* are endophytes, which are microbes grow inside the plant tissues without causing any harm to the host [35, 36]. Endophytes play an important role in improving stress tolerance of the host because they can produce active materials, fix nitrogen, accelerate plant to grow, and enhance the immune system and allelopathy of the host [37]. *Lysobacter* strains efficiently colonize on the root surfaces of several plants, including spinach, tomato, *Arabidopsis thaliana*, and *Amaranthus gangeticus* [38]. Thus, plant-parasitic nematodes have long-term co-existence with endophyte or root-colonized *Lysobacter* species in the plant host. Bacteria frequently respond to selective pressures and adapt to new environments by acquiring new genetic traits from other species via genetic communication. Therefore, genetic communication of *far* genes might have occurred between bacteria and plant-parasitic nematodes.

### Duplication, genus-level expansion, and distinct ligands binding of FAR in Clade V nematodes

**Fig. 5:**
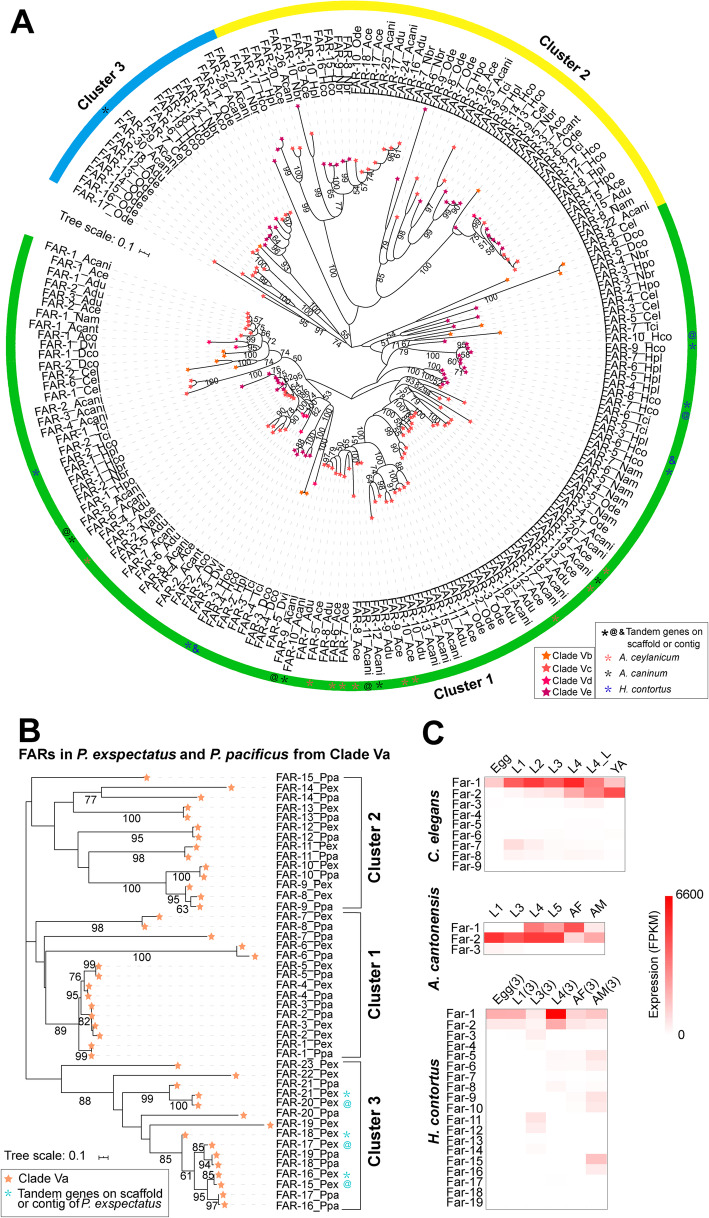
Evolution of FAR in Nematodes from Clade V. **A** Phylogenetic tree of FAR domain sequences among species from Clades Vb, Vc, Vd, and Ve. FAR proteins are separated into three clusters in the tree. Bootstrap values are shown in the nodes. The scale bar represents the number of amino acid substitutions per site. The green, yellow, and blue blocks on the ring represent the clusters 1, 2, and 3, respectively. The gene name includes abbreviated species name, as depicted in Fig. 1. The star with different colors indicates different subclades. *, @ and & indicate tandem replication of *far* gene on scaffolds or contigs, respectively. **B** Phylogenetic tree of FAR domains in two *Pristionchus* species from Clade Va. Bootstrap values are shown in the nodes. The scale bar represents the number of amino acid substitutions per site. The orange star on the branch represents FAR proteins from Clade Va. **C** Expression pattern of *far* genes in developmental stages of *C. elegans*, *A. cantonensis*, *H. contortus* with available RNA-seq data. Detailed expression values are shown in Additional file 4: Table S3. L4_L: later fourth-stage larvae, YA:young adult, AF:adult female, AM:adult male

Phylogeny analysis of 221 orthologs of FAR from free-living and parasitic nematodes in Clade V identified a *Pristionchus-*specific group. Among the 21-23 *Pristionchus far* genes in the genomes, 17 were placed in this group (Additional file 1: Fig. S13). This suggests that *far* genes from *Pristionchus* experienced lineage-specific duplications and these duplicated orthologs had low sequence homology to the orthologs in other species from Clade V. We further analyzed the phylogenetic relationship of FAR orthologs in Clade V without *Pristionchus*. Orthologs of FARs could be separated into three main clusters (Fig. 5). In free-living nematodes, nine FARs encoded in the genome of *C. elegans* were placed in three clusters, with seven of them in cluster 1. The tandem duplicated *C. elegans far-1* and *far-2* are located in chromosome III and have intron splice sites similar to *C. elegans far-6*. Tandem *C. elegans far-*3, -4, and -5 located in chromosome V also have similar intron splice sites and were clustered together in phylogenetic analysis (Additional file 1: Fig. S14). *C. elegans* FAR-7 was placed in cluster 3; and the novel *C. elegans* FAR-9 was placed in cluster 2. Results of the analysis of RNA-seq data showed that *far-1* and *far-2* genes of *C. elegans* had higher expression than others across developmental stages (Fig. 5 and Additional file 4: Table S3). In contrast, the 6 FARs encoded in the genome of *D. coronatus* were all placed in cluster 1, forming three branches. Thus, the orthologs of FARs among free-living *Pristionchus*, *D. coronatus*, and *C. elegans* have diverged early and experienced independent duplications.

#### FARs are possibly important in parasitism of Strongylida

Members of Strongylida are a large group of animal-parasitic nematodes residing in the intestine, respiratory tract, blood vessels, and other sites of host. In Strongylida, FARs obviously have experienced expansions in intestinal parasitic-nematodes from Clades Vc and Ve, including hookworms *Necator americanus* (8 copies, Clade Vc) and *Ancylostoma* (18-30 copies, Clade Vc), nodule worm *Oesophagostomum dentatum* (17 copies, Clade Vc), and strongylids *Nippostrongylus brasiliensis* (12 copies, Clade Ve) and *Haemonchus* (12-19 copies, Clade Ve)*.* These FARs were placed in three clusters and some subclusters. Approximately 44% of the expanded FARs were clustered into a specific group within cluster 1 (Fig. 5). Gene locus analysis showed that some expanded *far* genes in *A. ceylanicum*, *A. caninum*, and *H. contortus* were in tandem (Fig. 5 and Additional file 1: Fig. S15). Phylogenetic analysis indicated that FARs from intestinal parasitic nematodes in Clades Vc and Ve formed at least 6 monophyletic groups. Analysis of ES proteins indicated that the *far* genes in *O. dentatum* are transcribed at the high level in parasitic stages (L4 and adults) [39]. In our analysis, the *H. contortu*s *far* genes had the highest expression in L3, L4, and adult. In addition, *H. contortus far-1* and *far-2* had stage-specific expression and were expressed at the higher levels than other orthologs across developmental stages (Fig. 5 and Additional file 4: Table S3). Similarly, *N. americanus far-1* gene is known to have abundant expression across developmental stages [40]. In lungworms, orthologs of FAR in murine *Angiostrongylus* and bovine *D. viviparus* from Clade Vd were limited to 3–4. The FARs of two *Angiostrongylus* species clustered together and formed three subclusters. In contrast, FARs from *D. viviparus* under the superfamily of Trichostrongyloidea were placed in two branches within cluster 1. In *A. cantonensis*, *far-1* had the highest expression in parasitic stages (L4 and female) in the definitive host rat, *far-2* had high expression in L1 and L3, which are larval stages in the intermediate host snail (Fig. 5 and Additional file 4: Table S3), while *far-3* had low expression across developmental stages. In *D. viviparus*, *far-1* had higher expression in juveniles and adults than other developmental stages, while *far-2* had high expression in all stages from eggs to adults. Thus, FARs in both lungworms had divergent sequence and gene expression patterns, with higher *far-1* and *far-2* expression across developmental stages than others.

**Fig. 6:**
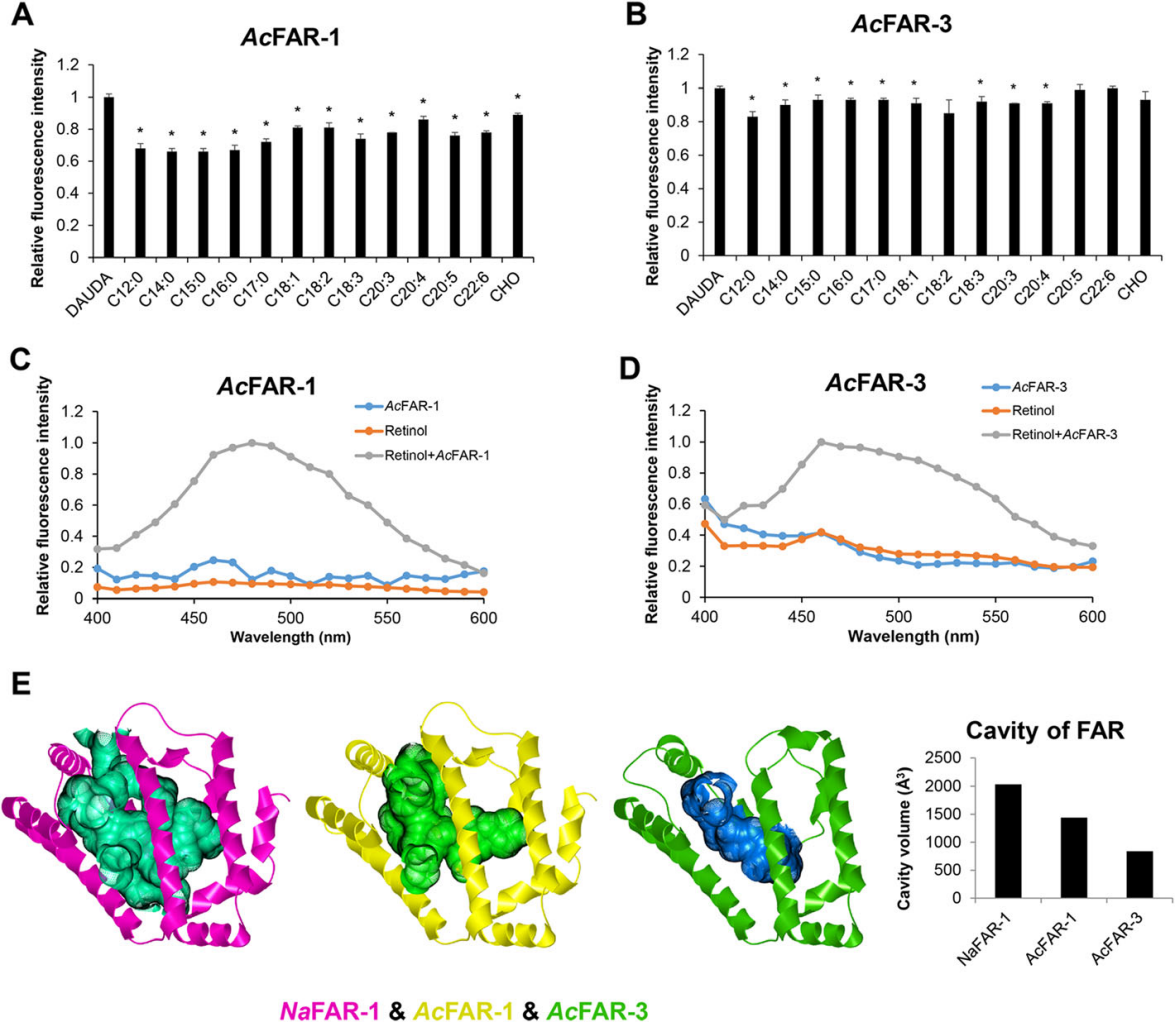
Ligand-binding Ability of *A. cantonensis* FAR Proteins. **A**, **B** The binding ability of *A. cantonensis* FAR-1 and FAR-3 to fatty acids and cholesterol. * *p <0.05*. C12:0–C17:0, saturated fatty acids with different carbon chains; C18:1, octadecenoic acid; C18:2, linoleic acid; C18:3, linolenic acid; C20:3, eicosatrienoic acid; C20:4, arachidonic acid; C20:5, eicosapentaenoic acid; C22:6, docosahexaenoic acid; CHO, cholesterol. **C**, **D** The binding ability of *A. cantonensis* FAR-1 and FAR-3 to retinol. **E** Comparison of the cavity volume of *N. americanus* FAR-1 (*Na*FAR-1) (PDB ID:4XCP), *A. cantonensis* FAR-1 (*Ac*FAR-1), and FAR-3 (*Ac*FAR-3)

FAR is a lipid-binding protein and is involved in the transport of fatty acids and retinol to modulate cell growth and proliferation. Subcellular localization analysis indicated that most FAR proteins were secretory proteins containing signal peptide (406/586) (Additional file 2: Table S1). Recent studies of plant-parasitic nematodes indicated that secretory FAR-1 s are localized in the hypodermis of nematodes [17, 18]. Functional *C. elegans* FAR proteins have distinct abilities of binding fatty acids and retinols. *C. elegans* FAR-1 through -6 can bind fatty acids and retinol, but FAR-7 has weak binding capacity for 11-(5-dimethylaminonaphthalene-1sulfonyl amino) undecanoic acid (DAUDA), retinol, and C18:4 [41, 42]. FAR-1 proteins in parasitic nematodes are known as functional proteins that bind fatty acids and retinol (Additional file 6: Table S6) [21, 41–45]; however, there is a lack of information on the ligand binding ability of other FARs with low sequence identity to FAR-1. We cloned *far*-1 and *far*-3 genes of *A. cantonensis* to assess the ligand binding ability of FARs with low sequence identity*.* In fluorescence-based ligand-binding assays, *A. cantonensis* FAR-1 bound the fluorescent fatty acid analog DAUDA and naturally fluorescent retinol (Fig. 6C and Additional file 1: Fig. S16). The degree of blue shift in DAUDA fluorescence emission (from 550 nm in buffer to 525 nm) indicated that FAR-1 had a highly apolar binding ability, as described for FAR-1 from other species [22, 46]. The preference of FAR-1 for fatty acids was investigated through the addition of fatty acids with different chain lengths in the DAUDA assay. DAUDA displacement occurred with fatty acids ranged C12:0–C22:6, especially the saturated C15:0 (Fig. 6A). The results obtained suggested that *A. cantonensis* FAR-1 had binding ability with retinol (Fig. 6C), while *A. cantonensis* FAR-3 had weak binding ability with fatty acids and retinol (Fig. 6B and 6D), which is similar to the function of *C. elegans* FAR-7 [42]. Further structural analysis revealed that *A. cantonensis* FAR-1 and FAR-3 were α-helix-rich proteins that closely resembled FARs from other nematodes [41, 43]. They had typical binding pockets as *N. americanus* FAR-1. The cavity volume of *A. cantonensis* FAR-1 was 1437.6 Å^3^, which is smaller than 2031.5 Å^3^ in *N. americanus* FAR-1, but significantly bigger than 836 Å^3^ in *A. cantonensis* FAR-3 (Fig. 6E). Thus, the differences in sequences and protein structures might lead to differential ligand-binding properties of FAR proteins.

## Discussion

Nematodes are helminths with diverse in lifestyles. Nematode FAR proteins are a family of lipid-binding proteins with divergent numbers and sequences [42, 47]. In this study, we have attempted to use combined data on the structure, phylogeny, expression patterns, and ligand-binding properties of nematode *far* genes to elucidate their complex evolutionary history.

### Lineage-specific expansion, duplication of FARs in free-living nematodes

Free-living nematodes commonly feed on bacteria, algae, fungi, dead organisms, and living tissues. They are usually the most abundant type of nematodes in soil and marine environments. It has been estimated that more than half of the nematode species existing are free-living. Molecular comparative studies indicated the existence of divergence and independent evolution of FARs among limited free-living nematodes. In *C. elegans*, tandem duplications have led to the presence of 9 *far* genes. In *Pristionchus*, intra-genus analysis of phylogeny showed that orthologs of FARs formed 3 clusters, which was also observed in other nematodes. However, 17 of the over 20 *far* genes in two *Pristionchus* species formed a single cluster in phylogeny analysis of Clade V. Although *Pristionchus* nematodes are regarded as a sister group of some rhabditids because of their similarity in morphology, culture conditions, and mode of reproduction, they have a close association with scarab beetles and the Colorado potato beetle and intercept the beetle’s sex communication system for host recognition. This probably has led to a lineage-dependent differentiation from rhabditids [48, 49]. Genome analyses of *C. elegans* and *Pristionchus* species indicated that both had substantial differentiation and only shared limited microsynteny; most conservation signals had vanished at the nucleotide level, and the proteins had an average 60% sequence identity [50, 51]. These data support the contention that the *far* orthologs of *Pristionchus* have diverged from their common ancestor of Clade V and undergone the lineage-specific expansions to adapt their lifestyle. *D. coronatus*, a close relative of *C. elegans*, have six FARs with relatively high sequence identity, forming three branches in cluster 1. *D. coronatus* reproduces parthenogenetically, consists of two chromosomes (2n = 2), and shares 59% sequence homology with *C. elegans*. A number of genes involved in sex determination and meiosis are missing or very divergent in *D. coronatus* [52]. Reproduction modes and lifestyles have apparently induced genomic changes, which might be responsible for the divergence of FARs among free-living *D. coronatus*, *Pristionchus* species, and *C. elegans*. Thus, the evolution of FARs in free-living nematodes could be influenced by reproduction mode or adaptation to ecological niches. More genomes of free-living nematodes will be needed in elucidating the evolutionary features of FARs.

### *Far* genes represent genus-wide diversity in animal-parasitic nematodes

Animals and humans are affected by parasitic nematodes from orders Ascaridida, Spirurida, Rhigonematida, Oxyurida, and Strongylida in Clades III, IV, and V. In our study, FARs have shown genus-level diversity in gene copies and sequences in parasitic nematodes of Clades III, IV, and V.

#### Ascaridida species have independent evolution of far genes

In Clade III, FARs in Oxyuridae, Ascaridida, and Spirurida formed three clusters in phylogenetic analyses of sequences. Only FARs from three Ascaridida species in Clade IIIb are placed in two branches in cluster 2. In addition, FARs from Ascaridida species have different length and numbers of introns compared with those from Oxyuridae and Spirurida. Results of comparative analysis of mitochondrial genomes have supported the suggestion that Oxyurida and Spirurida are sister groups to the Ascaridida and Rhabditida clades, suggesting that zooparasitic nematodes represented by Ascaridida, Oxyurida, and Spirurida do not share a recent common ancestor [53]. Thus, we proposed that Ascaridida has been separated from the ancestor of Oxyurida and Spirurida early and has experienced an independent evolution. Moreover, this evolutionary relationship of Oxyuridae, Ascaridida, and Spirurida is reflected by the evolution of the *far* genes.

#### Genus-wide expansion of far genes in Clade IVa and IVb

Orthologs of FARs have genus-wide expansion in *Steinernema* from Clade IVa and *Strongyloides* from Clade IVb. The FAR domain is one of the 20 most abundant Pfam domains present in the *Ste. carpocapsae* genome. Further analysis has revealed that *Steinernema* FARs formed at least 14 monophyletic groups through tandem duplication. Some *Steinernema far* genes have high expression in egg or L1, but others have high expression in L3 [20]. Previous studies proposed that the expanded FARs in *Steinernema* might play a key role in parasitism by regulating host retinoids in immune evasion or suppression [20]*.* Lineage-specific expansion of *far* genes in *Steinernema* and the relatively higher expression of the genes in infective or parasitic stages may be beneficial to the parasitism of *Steinernema*. FARs in Strongyloididae have also formed at least 14 monophyletic groups. *Strongyloides* species, however, have intron loss in *far* genes, with similar to other genes in the genome level. Analysis of RNA-seq data from parasitic adult female (simplify as P_AF) and free-living adult female (simplify as F_AF) has shown higher expression of *far-1* and *far-2* in adults than other *far* genes [29–31]. These data suggest that the *Steinernema* and *Strongyloides* lineages most likely have not inherited these expanded *far* genes from a common ancestor. Instead, we proposed these genes have undergone separated amplifications in both lineages in adaptation to parasitism.

#### Variable richness and low sequence homology of FARs in Strongylids

The evolutionary relationship of FARs in different species from Clade V is not consistent. Species in Clades Vc and Ve are intestinal parasites of Strongylida. FARs in these intestinal parasitic-nematodes have gone through expansions, forming at least 6 monophyletic groups. However, the number of *far* genes in lungworms of Clade Vd is less than 5, which is consistent with the median in 58 nematodes. FAR sequences in murine *Angiostrongylus* species and bovine *D. viviparus* (a member of the Trichostrongyloidea) are divergent. Both lungworms separated from each other more than 300 million years ago and evolved distinct life cycle. *Angiostrongylus* uses an indirect life-cycle and needs an intermediate host of slugs or snails that ingests L1. In contrast, *D. viviparus* has a direct life cycle with L1 developing in feces into infective L3 to infect a new host. L3 passes through the alimentary tract, penetrates the wall of the intestine, and migrates to the lungs. A high expression of *far-1* and *far-2* in parasitic stages (L4 and adults) was found in the analyses of transcriptome data from both intestinal parasitic nematodes and lungworms. FAR-1 and FAR-2 proteins have high expression level in both parasitic stages (L4 and adults) in *O. dentatum* [39]. Sequence divergence and variations in copy numbers of *far* genes in Strongylida can be attributed to the adaptative evolution in intestinal parasitic-nematodes with *far* genes undergoing tandem duplications and in lungworms with *far* genes experiencing sequence divergence.

### Lineage-specific evolution of *far* genes in plant-parasitic nematodes

The number of *far* genes in root-knot nematodes might relate to the reproduction mode of facultative meiotic parthenogenesis and obligatory mitotic parthenogenesis. *Far-1* of root-knot nematode *Pratylenchus penetrans* has high expression in juveniles, adult females, and males. Suppressing the expression of *P. penetrans far*-1 by RNAi significantly reduced the reproduction of nematodes [17]. Thus, we propose that *far* genes in root-knot nematodes might originate from one ancestor, and FARs can regulate the reproduction of these organisms. The gene number appears to be influenced by the reproduction mode. In cyst nematodes, gene duplications are present in *G. pallida* and *H. glycines.* In pinewood nematodes, lineage-specific duplications are seen in *Bursaphelenchus.* The silencing of Mj-FAR-1 in tomato hairy roots leads to the expression of a complementary dsRNA and reduced infection, while over-expression of the *far* gene can increase the infectivity of nematodes [25]. Jasmonic acid plays an important role in plant responses to biotic and abiotic stress [54, 55]. FAR proteins could inhibit the defense reactions of the host plant by obstructing gene expression of jasmonic acid pathway, and therefore play critical roles in the development and infection of plant-parasitic nematodes [22, 24]. Thus, the diversity in parasitism of plant-parasitic nematodes is consistent with variations in the richness and sequences of *far* genes. As a result, the evolution of *far* genes to some extent reflects the evolutionary dynamic of plant-parasitic nematodes.

### Evidences of horizontal gene transfer (HGT) of *far* genes from plant-parasitic nematode to bacteria

FARs are widely known as nematode-specific proteins. Our searches of the helminth genomes of *Schistosome mansoni*, *Echinococcus multilocularis*, and free-living *Schmidtea mediterranea* have identified no Gp-FAR-1 domains in these non-nematode species. However, we found one or two FAR proteins in five *Streptomyces* species, three *Lysobacter* species, *Bacillus hwajinpoensis*, and *Kitasatospora* sp. There is colinearity of FAR domains between plant-parasitic nematodes and bacteria, producing a similar set of sequences from *Streptomyces* and *Lysobacter* and some plant-parasitic nematodes. Plants are the host of many microbial endophytes, including bacteria, fungi, archaea, and parasitic nematodes [36]. *Bacillus* sp. is one of the dominant endophyte in *Bursaphelenchus* [35, 56, 57]. Fossil records of endophytes in plants have dated back to more than 400 million years, indicating that these microorganisms have co-evolved with hosts for millions of years [58]. The presence of endophytes *Streptomyces*, *Bacillus hwajinpoensis*, and *Kitasatospora* sp. promotes plant growth, elicits plant defense response against pathogens, and acts as remediators of abiotic stresses [37]. In addition, root-colonizing *Lysobacter* species can secrete a variety of antibiotics, extracellular hydrolases, and bio-surfactants to inhibit the growth of pathogens, controlling plant diseases [38]. Bacteria and plant-parasitic nematodes occupy similar niches in the soil and roots. We propose that the cohabitation in host tissues allows bacteria and plant-parasitic nematodes gaining special biological functions from each other through HGT.

HGT occurs frequently among prokaryotes and could also be found between eukaryotes and prokaryotes that occupy similar niches. *Pseudomonas aeruginosa* pldA might have acquired horizontally from a eukaryotic organism because it is homologous to PLDs from mammals and yeast [59]. Although the frequency of HGT between nematodes and bacteria is low, it could still be an important factor in the evolution of nematode parasitism. Gene sequences of β-1,3-glucanase in *B. xylophilus* have higher identity to bacteria than to eukaryotes, and no similar sequences are present in *C. elegans* and *C. briggsae*. *B. xylophilus* depends on secreted β-1,3-glucanase to degrade glucan in fungal cell wall that they feed on. It has been suggested that the gene may have been obtained from bacteria through HGT [60]. A recent comprehensive genomic analysis of *Legionella pneumophila* has revealed a surprising number of eukaryotic-like genes arisen via HGT from eukaryote [61]. In plants, the jasmonic acid signaling mediates resistance against necrotrophic pathogens [62]. FARs of plant-parasitic nematodes could counter the defense reactions of the host plant by reducing the expression of genes in the jasmonic acid pathway [22, 24]. Bacteria that have acquired the FARs could gain the ability to resist the biotic and abiotic stress in plants. Thus, we hypothesized that plant-parasitic nematodes may be the original carriers of the *far* genes and have passed them to some endophytes and root-colonized bacteria. Living in the same environmental niche could facilitate the transfer of beneficial genes into the genome. While it is likely that bacteria might have obtained *far* genes from associated plant-parasitic nematodes to defend against the jasmonic acid pathway of host plants, the underlying mechanism needs further studies. In codon usage analysis, the codon index of bacterial *far* genes is similar to that of the whole genome. This could be because the *far* genes in the bacterial genomes could have acquired a long time ago. Whether the transferred *far* genes function the same in binding and transporting chemotactic molecules to regulate chemotaxis need further studies.

### Possible biological function of FARs in parasitism

Most FAR proteins are secretory proteins and could be found in ES proteins of nematodes. The *far-1* and *far-2* genes have high expression in infective L3, L4 larvae, and adults in *T. canis*, *Stronglyloides*, and members of Strongylida. Although *C. elegans* FAR-7 and *A. cantonensis* FAR-3 have weak ability to bind fatty acids or retinols, nematode FAR-1 proteins can bind fatty acids and retinols and are involved in transport of fatty acids and retinol from host tissues to modulate cell growth and proliferation. It has been suggested that secretory FAR-1 interacts with eicosanoids-fatty acids to sequestrate host retinoids for immune evasion [24]. In plant-parasitic nematodes, FARs inhibit the defense reactions of the host plant by suppressing gene expression in the jasmonic acid pathway [22, 24]. As parasitism has arisen independently multiple times among nematodes [28], it could be speculated that FAR proteins have experienced multiple expansions and divergence to adapt to parasitism of plants, invertebrates, and vertebrates across the nematode lineages.

### The origin of FARs in nematodes

In our analysis, *far* genes have not been found in Trichocephalida and insect-parasitic nematode *R. culicivorax* from Clade I. Species in Clade I belong to Enoplea in the taxonomy, while those in Clades III, IV, and V belong to Chromadorea. The ancestor species of Enoplea separated early from the common ancestor of Chromadorea. In addition, species of Enoplea have very different patterns in the early cell division and cell fate assignment compared with species in Chromadorea [63]. Species in Clades III, IV, and V from Chromadorea vary in the features of FAR orthologs. As there are no information of FARs in nematodes from Clade II, we propose two possibility of the ancestral origins of nematode FARs. One possibility is that *far* genes might have originated from the common ancestor of nematodes in Clades III, IV, and V. Another is that *far* genes might be originated from ancestor of phylum Nematoda but were lost in the ancestor of Clade I. Currently, the second hypothesis is less convincing.

## Conclusions

In summary, tandem duplications and lineage-specific expansions apparently have led to genus-wide expansions of *far* genes in some nematodes. The variable richness and low homology of *far* genes further indicate that *far* genes have diverged early and experienced low selective pressure in adaptation to parasitism of plants, invertebrates, and vertebrates in the evolutionary process. Extensive analyses of bacterial *far* genes have provided the evidence that nematode *far* genes might have been transferred to cohabitating bacteria, which need further functional studies. These observations provide new insights into the biology of FAR proteins and indicate that the FAR gene family potentially represents a rich source of data for improved understanding of nematode evolution.

## Methods

### Genome-wide identification of FAR proteins in nematodes and bacteria

Genome assemblies of 58 nematodes were retrieved from Wormbase WBPS10 [64]. We filtered fragmental genome according to assembly metrics and kept one high-quality assembly for multi-assembly species as described [34]. We employed the same pipeline to identify FAR proteins in nematode genomes [34]. We downloaded Gp-FAR-1 sequence from the Swissprot database as query in a homology search of the nematode genomes using HMMSEARCH with parameters 1e-3. Solar was used to join high-score blocks and GeneWise was performed to predict gene structure [65]. We also employed HMMER to detect potential FAR proteins in the original genome annotation. We filtered fragmental FAR protein with length less than 100 aa and manually examined gene numbers. The transmembrane domains, subcellular localization, and signal peptide in FAR proteins were identified using DASTMfilter, Cell-Ploc, and SignalP. We assessed sequence identity of FAR domain in observed 58 nematodes using Sias (http://imed.med.ucm.es/Tools/sias.html), and heatmaps of sequence identity of FAR domain generated using TBtools (v1.087). We used the Gp-FAR-1 domain as a query to search homologous sequences in 31,332 bacterial genomes in the Ensembl Bacteria database using BLAST (http://bacteria.ensembl.org/).

### Phylogenetic analyses

We performed comparative analyses to study the evolution of FAR proteins across the phylum Nematoda. Initially, we used MUSCLE to do multiple sequence alignment based on protein sequences [66]. Then, IQ-TREE (v1.6) and MEGA-X were employed to select the best model for Maximum-Likelihood or Neighbor-Joining analyses and reconstruct phylogenetic trees [67]. Visualization was achieved using evolview (https://www.evolgenius.info/evolview) [68]. We initially clustered 586 FARs into orthologous groups using OrthoMCL [69] and then reconstructed the phylogeny independently for nematodes in each clade.

### Expression profile analyses

To investigate expression pattern of FAR proteins in developmental stages, we downloaded RNA-seq data of 13 nematodes from the SRA database (Additional file 4: Table S3). FastQC (v0.11) was used to check the quality, and Trimmomatic (v0.38) was used to filter low-quality reads [70]. Thereafter, we mapped reads to the reference genome with HISAT2 (v2.1) [71]. We used featureCount of Subread package (v1.6) to obtain read count of *far* genes [72]. We used FPKM (Fragments Per Kilobase Million) or RPKM (Reads Per Kilobase Million) to normalize the expression of *far* genes for paired-end or single-end RNA-seq, respectively.

### Comparison of FARs from plant-parasitic nematode and bacteria

GC% of *far* genes and the other genes in the genomes of nematode and bacteria was calculated using Geneious (v2021.0.3) [73]. Gene structures of *far* genes were visualized using TBtools (v1.087) [74], and their collinearity was analyzed using CoGe’s Genome Evolution Analysis Tool with the TBlastX alignment algorithm (https://genomevolution.org/CoGe/GEvo.pl). We assessed sequence identity of FAR domains between nematodes and bacteria using Sias (http://imed.med.ucm.es/Tools/sias.html), and heatmaps of sequence identity among FAR domains were constructed using TBtools (v1.087). Codon usage analyses, including the codon adaptation index (CAI), codon bias index (CBI), effective number of codon (Nc), and frequency of optimal codons (FOP), were calculated using CodonW with the default parameters (1.4.4, http://codonw.sourceforge.net/). The genomic features and annotations of genes upstream and downstream of the candidate horizontal transfer genes were analyzed.

### Expression and purification of recombinant protein

Total RNA was extracted from adult *A. cantonensis* and reverse-transcribed into cDNA. *A. cantonensis far*-1 and *far*-3 cDNA were amplified by PCR and cloned into the pGEX-4 T-1 expression vector. Recombinant full-length protein without signal peptide was expressed in *E. coli* BL21 (DE3). The expression of the *A. cantonensis* FAR-1 and FAR-3 proteins was induced by incubation with 1 mM isopropylthio-β-galactoside (IPTG) at 37 °C for 6 h. The recombinant GST-FAR-1 and GST-FAR-3 were purified using GSTSep glutathione agarose resin (Yeasen, China). The GST tag was cleaved by incubation with thrombin enzyme (Meilune, China), resulting in the production of *A. cantonensis* FAR-1 and FAR-3. The purity of FAR-1 and FAR-3 proteins was assessed using sodium dodecyl sulfate-polyacrylamide gel electrophoresis.

### Fluorescence-based ligand binding assays

Fatty acid- and retinol-binding activities of recombinant *A. cantonensis* FAR-1 and FAR-3 proteins were measured using the fluorescent analogs DAUDA (Sigma, USA) as previously described [44]. DAUDA, retinol (Sigma, USA), and other fatty acids (Aladdin, Shanghai) were prepared as stock solution of 10 mM in ethanol. DAUDA and retinol were used at 1:100 dilutions in PBS, while other fatty acids were diluted at 1:10 in PBS. The protein concentrations of *A. cantonensis* FAR-1 and FAR-3 were calculated to be at approximately 1 mg/mL. Competition binding experiments were carried out as previously described [44]. Fluorescence emission spectra were recorded at 25 °C with a total volume of 150 μl per well in black 96-well microfluor 1 plates (Corning, USA) using a SpectraMax M5 (Molecular Devices, USA). The fluorescence emission spectra for FAR-1 and FAR-3 bound to DAUDA and retinol were determined in a similar manner. The excitation wavelengths used for DAUDA and retinol were 345 and 350 nm, respectively. All fluorescent compounds were stored at − 20 °C and freshly diluted in ethanol before use.

### Modeling of *A. cantonensis* FAR-1 and FAR-3

To examine the structural basis of *A. cantonensis* FAR proteins for ligand binding, the 3-dimensional models of FAR-1 and FAR-3 were established using SWISS-MODEL online with *N. americanus* FAR-1 (PDB ID:4XCP) as the template [75]. Although *A. cantonensis* FAR-3 shared relatively low protein sequence identity (22.8%) to *N. americanus* FAR-1, the homology modeling result of *A. cantonensis* FAR-3 was reserved as the reference model in further analyses. The cavity volume of each FAR protein was determined using CAVER 3.0 package [76]. Images of the structure were generated by PyMol viewer.

### Statistical analysis

Data were reported as the mean ± SD (standard deviation). The *t* test was used in the evaluation of differences between two groups. One-way ANOVA was used to assess the significance of the differences between groups, and *P <* 0.05 was considered statistically significant. The statistical analysis was performed using Prism 5.0 (GraphPad Software, CA).

## Supplementary Information

**Additional file 1: Figures S1 to S15.** FigS1 - Genus level changes in gene numbers of nematode FAR in different subclades. FigS2 - Sequence identity of FAR domain from nematodes in Clade III. FigS3 - Sequence identity of FAR domain from nematodes in Clade IV. FigS4 - Sequence identity of FAR domain from nematodes in Clade V. FigS5 - Protein Maximum Likelihood tree of FAR domain among nematodes. FigS6 - Gene structure of *far* from nematodes in Clade III. FigS7 - Maximum-Likelihood tree of 310 FAR proteins from nematodes in Clade IV. FigS8 - Tandem duplicated *far* gene in *Strongyloides ratti*, *Strongyloides stercoralis*, *Strongyloides papillosus*, *Steinernema carpocapsae*, *Steinernema scapterisci*, *Steinernema feltiae*, *Steinernema glaseri*, and *Rhabditophanes* sp. KR3021 from Clade IV. FigS9 - Gene structure and expression pattern of *far* in some plant-parasitic nematodes. FigS10 - Sequence identity and gene structure analyses of bacteria *far*. FigS11 - Genome colinearity in the CDS region of FAR domain between plant-parasitic nematodes and these bacteria. FigS12 - Genome colinearity in the CDS region of FAR domain among plant-parasitic nematodes, other nematodes from different clades, and these bacteria. FigS13 - Protein Maximum Likelihood tree of FARs from nematodes in Clade V. FigS14 - Intron analysis of tandem duplicated *C. elegans far.* FigS15 - Gene locus of tandem duplicated *far* gene in *Pristionchus exspectatus*, *Ancylostoma ceylanicum*, *Ancylostoma caninum*, and *Haemonchus contortus* from Clade V. FigS16 - Relative fluorescence intensity of *Ac*FAR-1 and *Ac*FAR-3 binding with DAUDA.

**Additional file 2: Table S1.** Gene information of FAR in reannotation and original gene annotation.

**Additional file 3: Table S2.** Comparison of gene and exon numbers of *far* in nematodes.

**Additional file 4: Table S3.** Transcriptome data information for 11 nematodes.

**Additional file 5: Tables S4 to S5.** Table S4 - GC content of plant-parasitic nematodes and bacteria. Table S5 - The five codon indices of FAR_CDS/all_CDS in bacteria.

**Additional file 6: Table S6.** Ligand binding ability of nematode FARs.

## Data Availability

The datasets analyzed during the current study are available in the public databases (Wormbase and NCBI). All relevant accessions of genomes and transcriptomes are listed in Additional file 2: Table S1 and Additional file 4: Table S3.

## Abbreviations

PUFAs: Polyunsaturated fatty acids
FAR: Fatty acid and retinol-binding protein
ES: Excretory-secretory
FPKM: Fragments Per Kilobase of transcript per Million mapped reads
RPKM: Reads per kilobase of gene per million mapped reads
HGT: Horizontal gene transfer
