# Supplementary material for: Genus-level evolutionary relationships of FAR proteins reflect the diversity of lifestyles of free-living and parasitic nematodes
Source: BMC Biol. 2021 Aug 30;19:178. doi: 10.1186/s12915-021-01111-3 (PMC8407040; doi:10.1186/s12915-021-01111-3)
Supplement: Supplementary file 1 — Additional file 1: Figures S1 to S15. FigS1 - Genus level changes in gene numbers of nematode FAR in different subclades. FigS2 - Sequence identity of FAR domain from nematodes in Clade III. FigS3 - Sequence identity of FAR domain from nematodes in Clade IV. FigS4 - Sequence identity of FAR domain from nematodes in Clade V. FigS5 - Protein Maximum Likelihood tree of FAR domain among nematodes. FigS6 - Gene structure of far from nematodes in Clade III. FigS7 - Maximum-Likelihood tree of 310 FAR proteins from nematodes in Clade IV. FigS8 - Tandem duplicated far gene in Strongyloides ratti, Strongyloides stercoralis, Strongyloides papillosus, Steinernema carpocapsae, Steinernema scapterisci, Steinernema feltiae, Steinernema glaseri, and Rhabditophanes sp. KR3021 from Clade IV. FigS9 - Gene structure and expression pattern of far in some plant-parasitic nematodes. FigS10 - Sequence identity and gene structure analyses of bacteria far. FigS11 - Genome colinearity in the CDS region of FAR domain between plant-parasitic nematodes and these bacteria. FigS12 - Genome colinearity in the CDS region of FAR domain among plant-parasitic nematodes, other nematodes from different clades, and these bacteria. FigS13 - Protein Maximum Likelihood tree of FARs from nematodes in Clade V. FigS14 - Intron analysis of tandem duplicated C. elegans far. FigS15 - Gene locus of tandem duplicated far gene in Pristionchus exspectatus, Ancylostoma ceylanicum, Ancylostoma caninum, and Haemonchus contortus from Clade V. FigS16 - Relative fluorescence intensity of AcFAR-1 and AcFAR-3 binding with DAUDA. [file 12915_2021_1111_MOESM1_ESM.docx]

**Supplementary Figures**


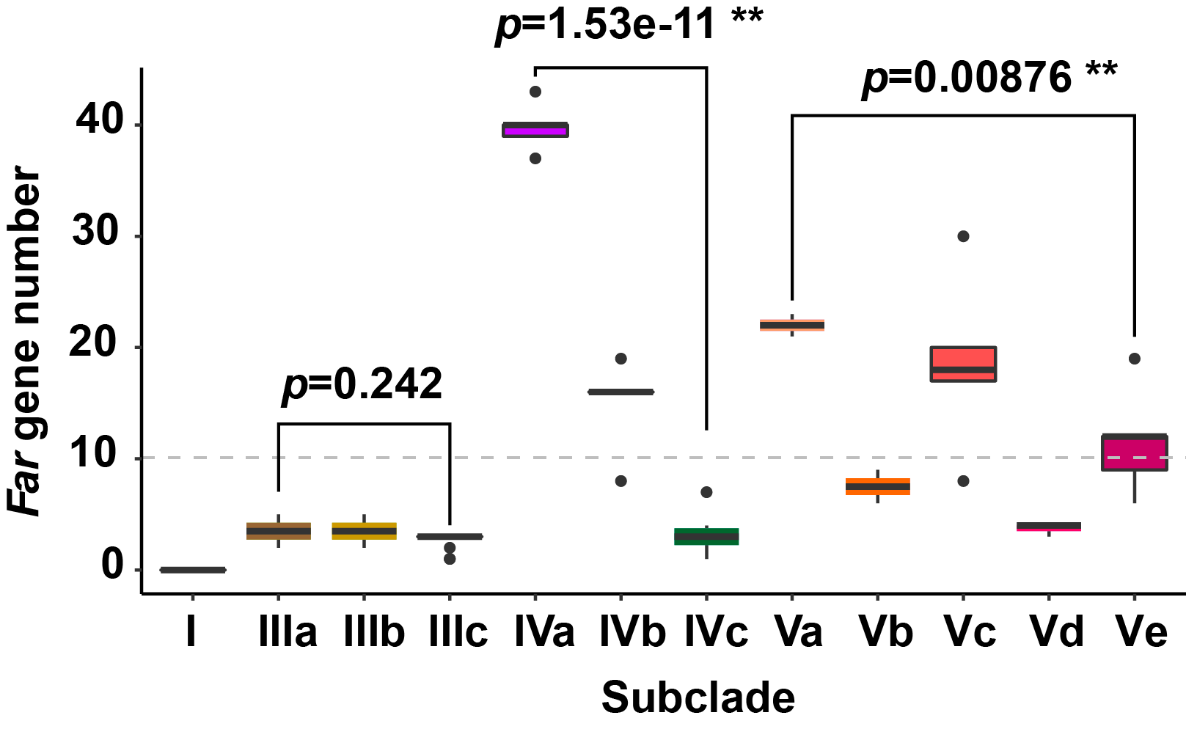


**Supplementary Figure S1** Genus level changes in gene numbers of nematode FAR in different subclades. Boxplots show the gene number distribution for *far* in each subclade. ** Intra-group variance *p*<0.01.


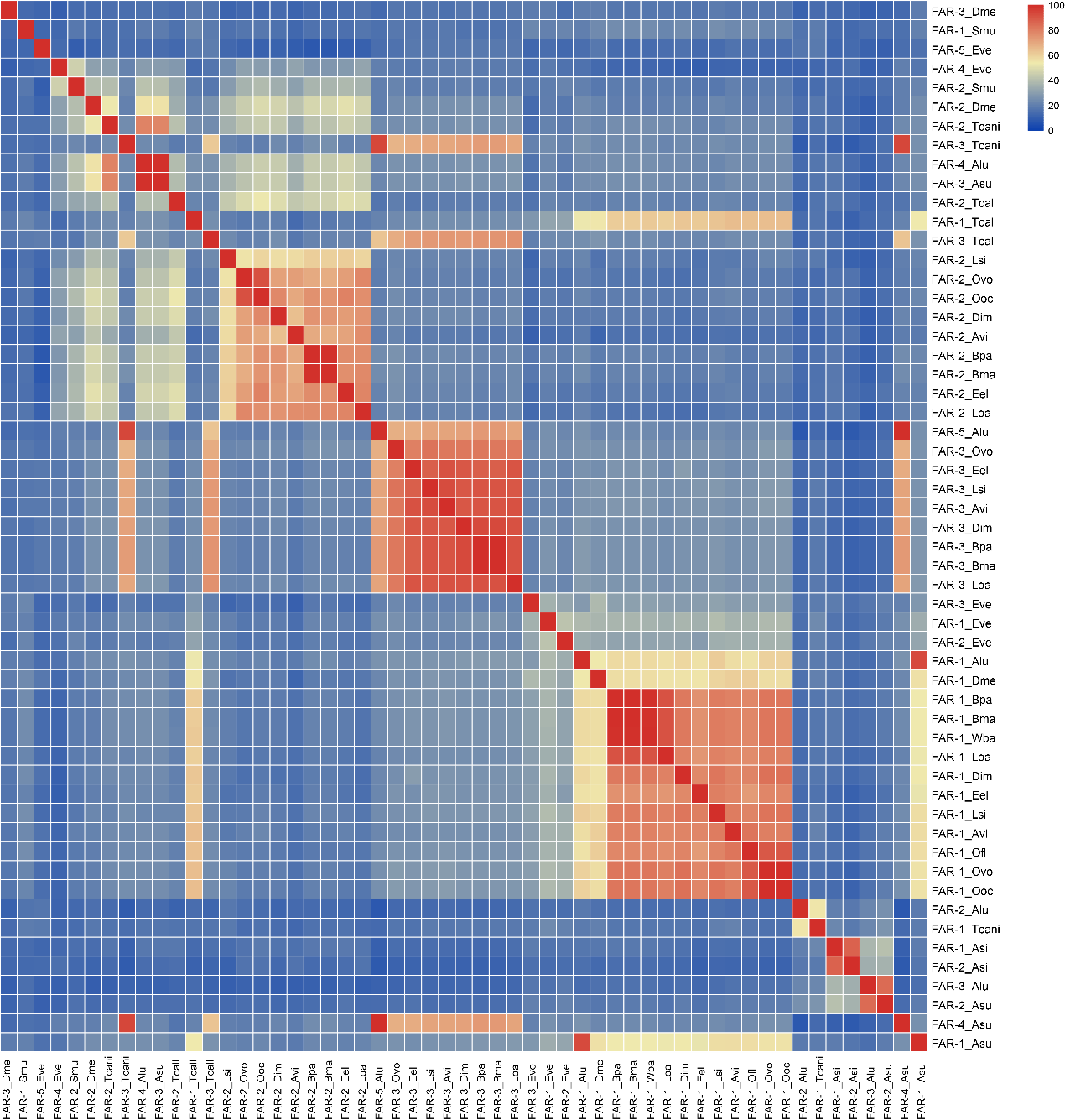


**Supplementary Figure S2** Sequence identity of FAR domain from nematodes in Clade III. Heatmap shows the percentage of sequence identity of both FAR domains from nematodes in Clade III.


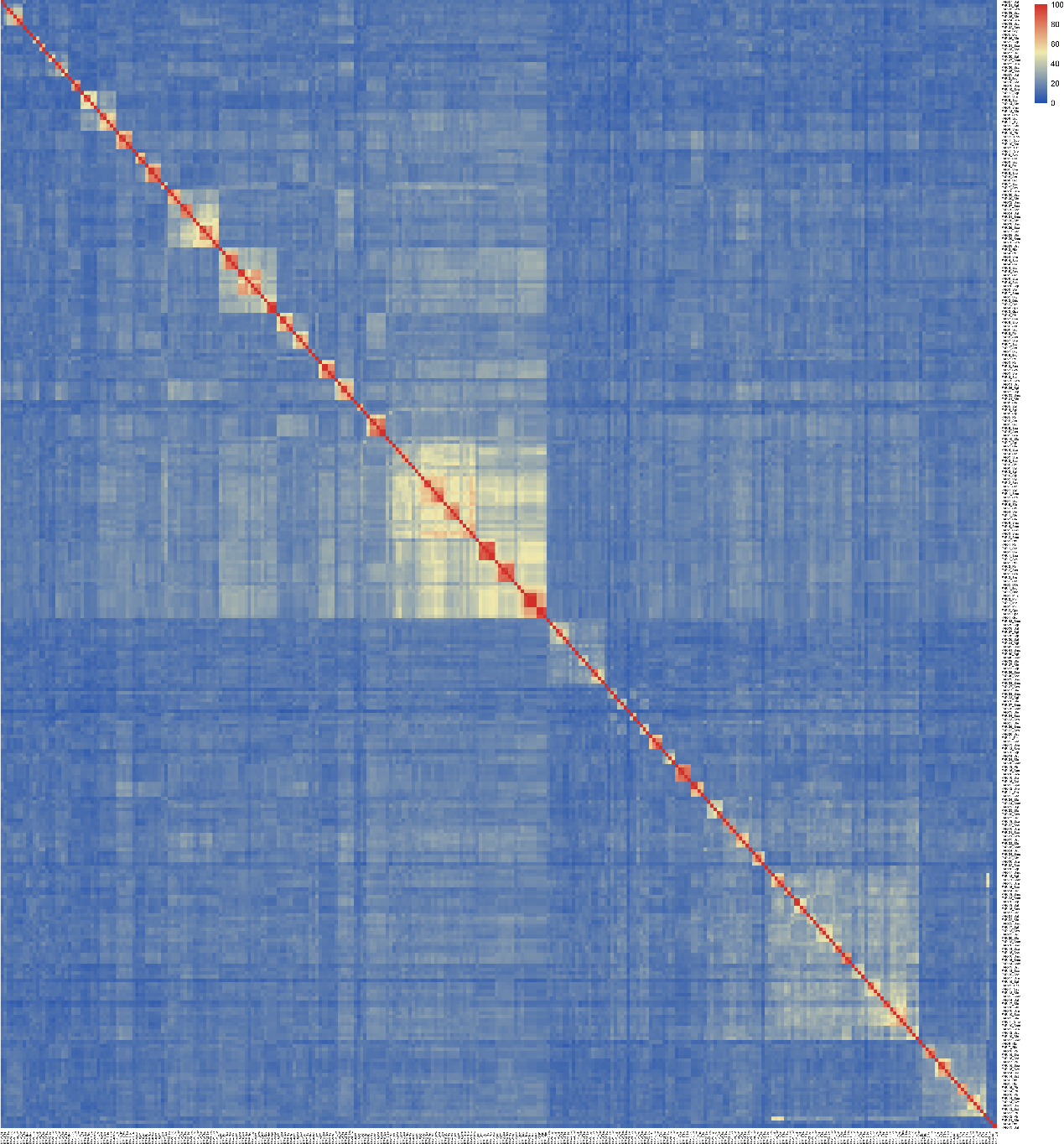


**Supplementary Figure S3** Sequence identity of FAR domain from nematodes in Clade IV. Heatmap shows the percentage of sequence identity of both FAR domains from nematodes in Clade IV.


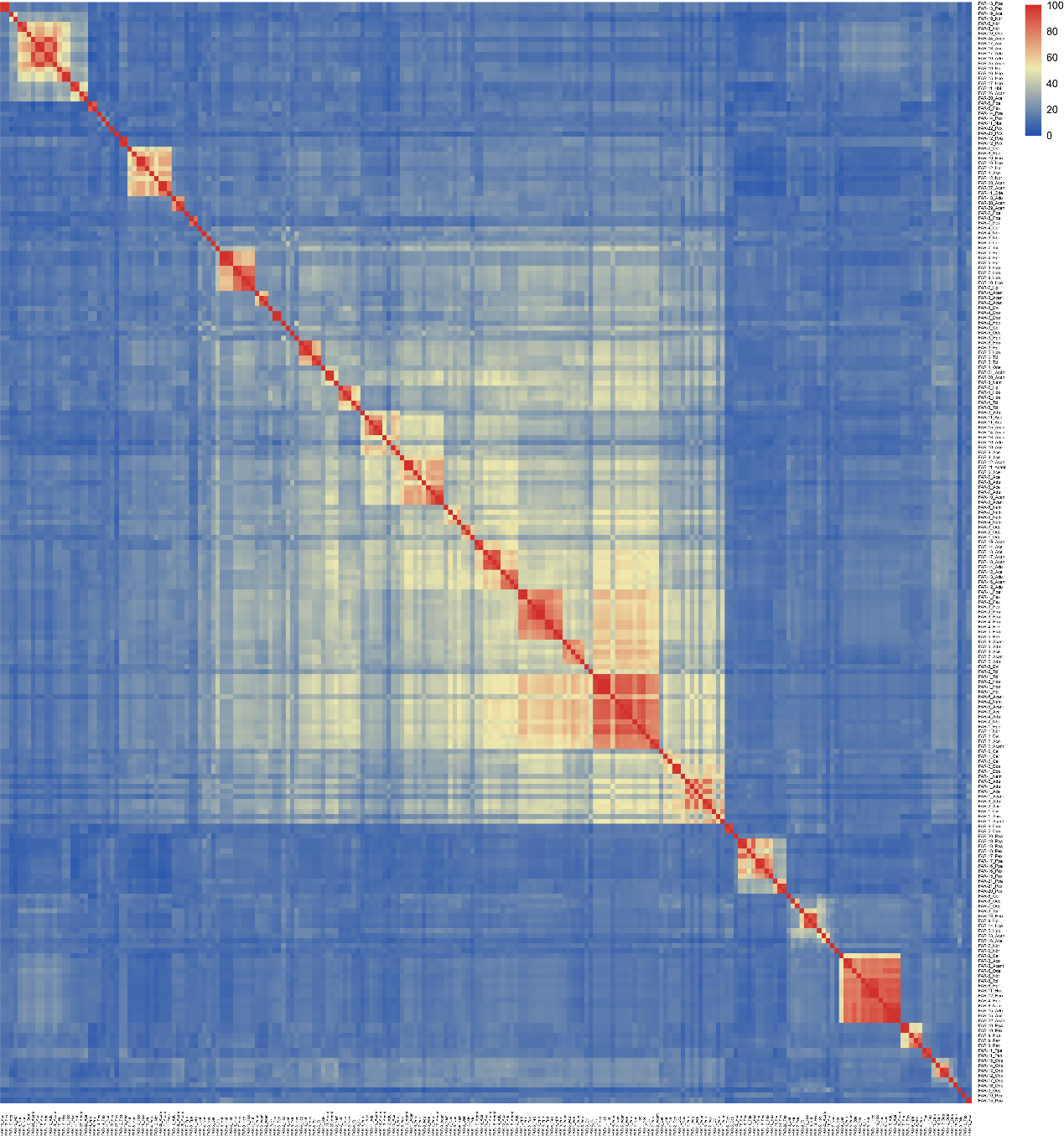


**Supplementary Figure S4** Sequence identity of FAR domain from nematodes in Clade V. Heatmap shows the percentage of sequence identity of both FAR domains from nematodes in Clade V.


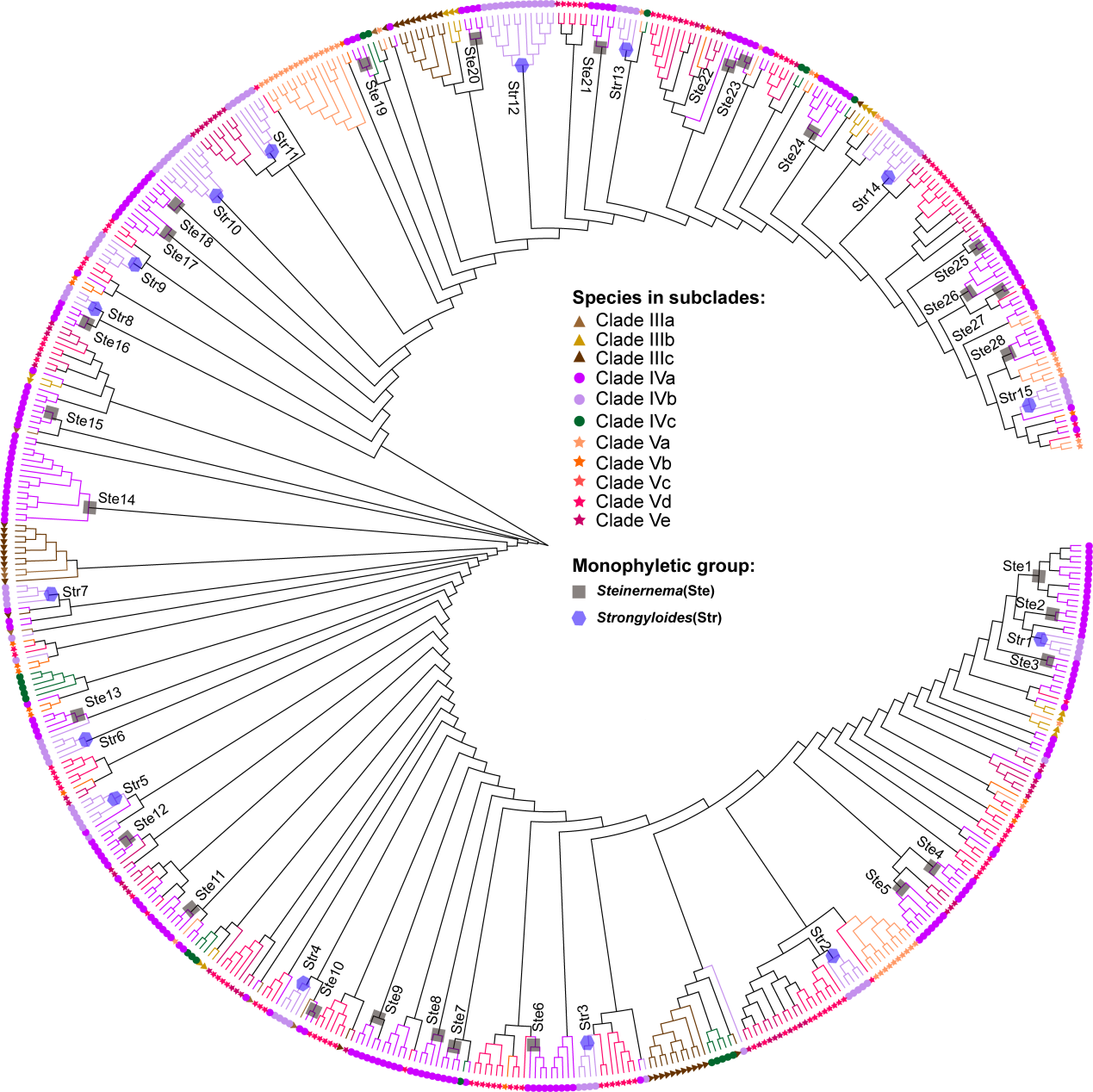


**Supplementary Figure S5** Protein Maximum Likelihood tree of FAR domain among nematodes. Total 586 FAR proteins from nematodes in phylogenetic tree were divided into different groups. Bootstrap values are shown in the nodes. The scale bar represents the number of amino acid substitutions per site. The deep grey square and blue-purple hexagon represent the monophyletic groups from *Steinernema* and *Strongyloides* species, respectively. The color of gene name on the ring is corresponding to the color of the branch. The designated shapes for clades and colors for subclades are used consistently throughout the study.


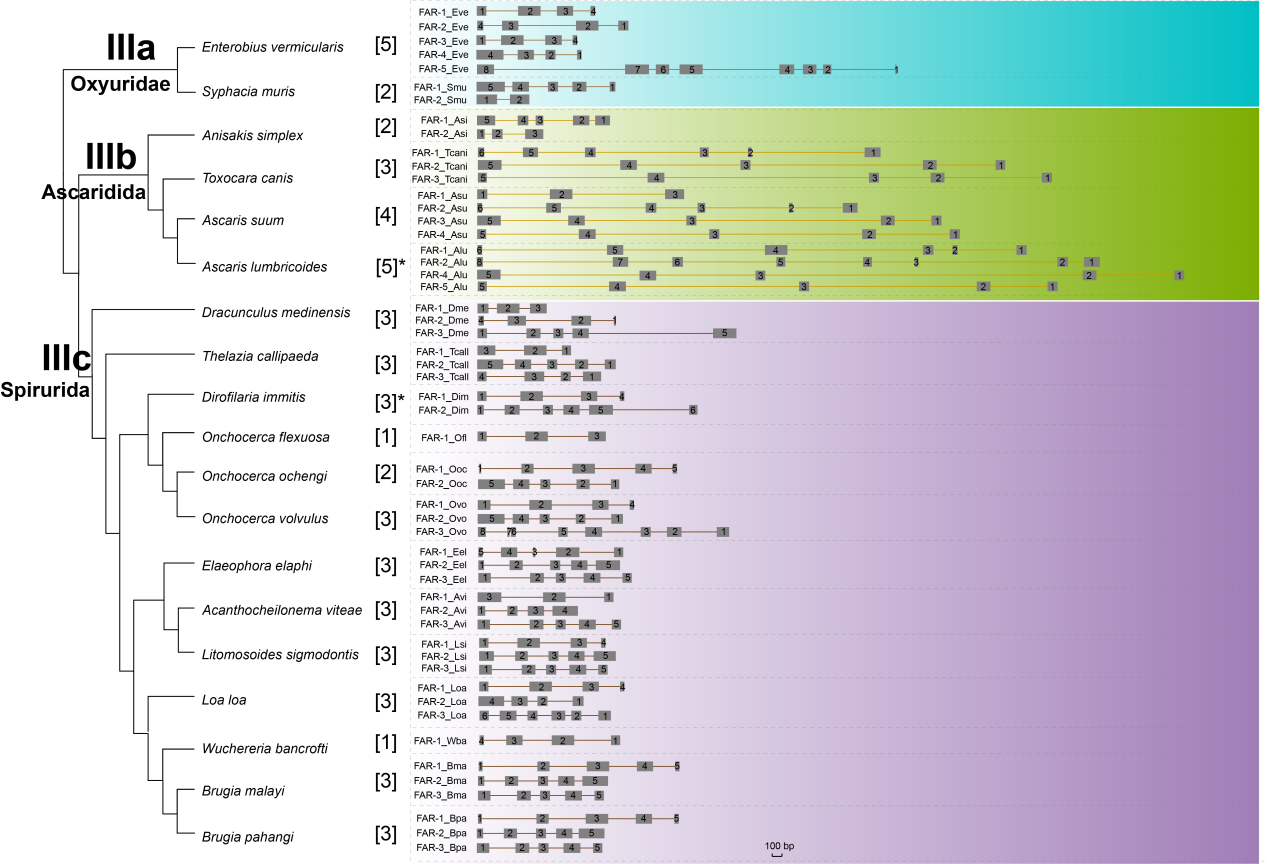


**Supplementary Figure S6** Gene structure of *far* from nematodes in Clade III. *genome assembly problem leads to *A. lumbricoides* *far*-3 and *D. immitis* *far*-3 hold together with other gene.

**
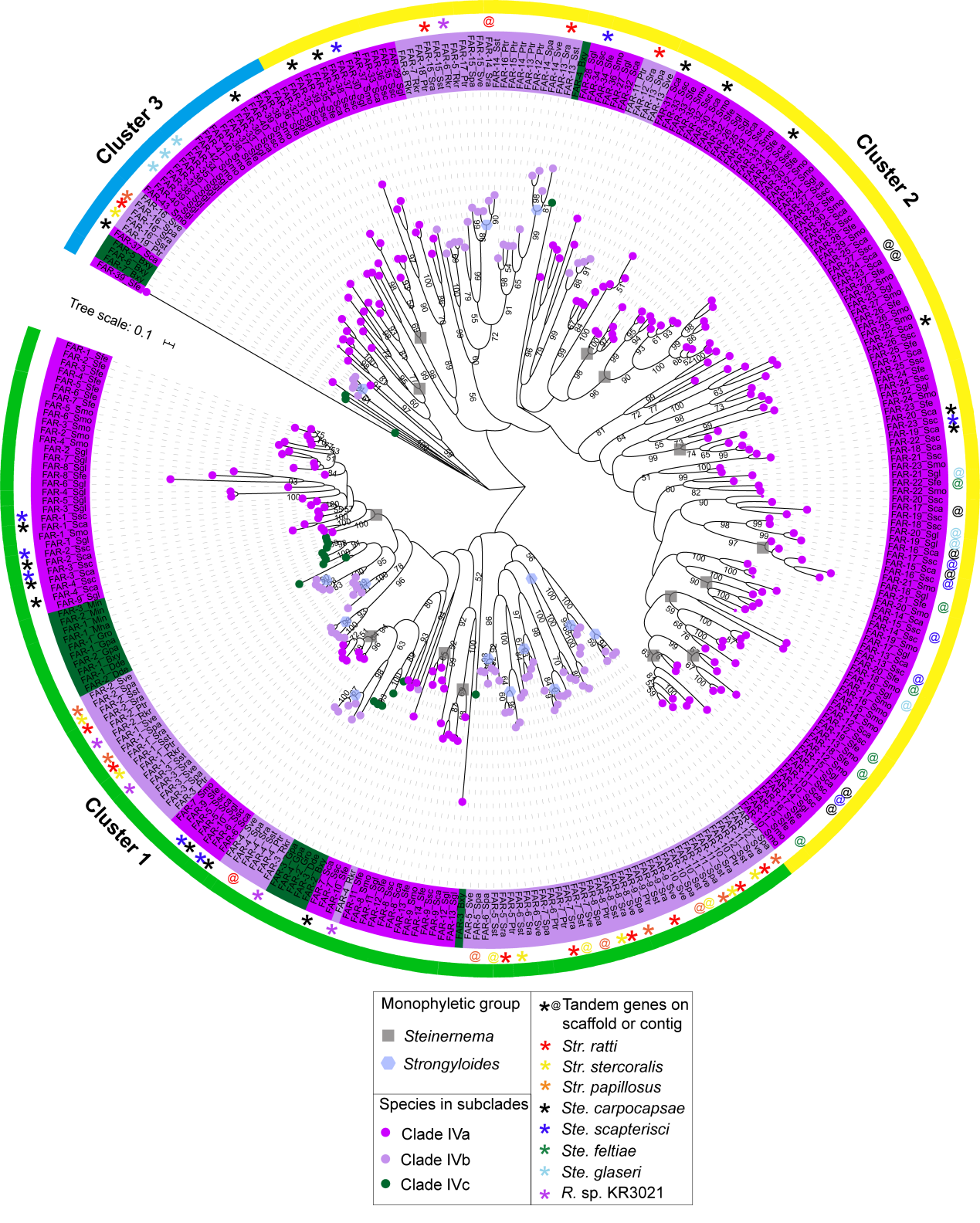
**

**Supplementary Figure S7** Maximum-Likelihood tree of 310 FAR proteins from nematodes in Clade IV. Bootstrap values are shown in the nodes. The scale bar represents the number of amino acid substitutions per site. The circles with different colors on the branches represent FAR protein from Clades IVa, IVb, and IVc. The background color of gene name on the inner ring is corresponding to the color of solid circle on the branch. The green, yellow, and blue blocks on the outer ring represent clusters 1, 2, and 3, respectively. The gene name includes abbreviated species name, as depicted in Fig.1 and Additional file 2: Table S1. The dark gray box and the blue purple hexagon represent the monophyletic groups of *Steinernema* and *Strongyloides*, respectively. * and @ indicate tandem replication on scaffolds or contigs, respectively.


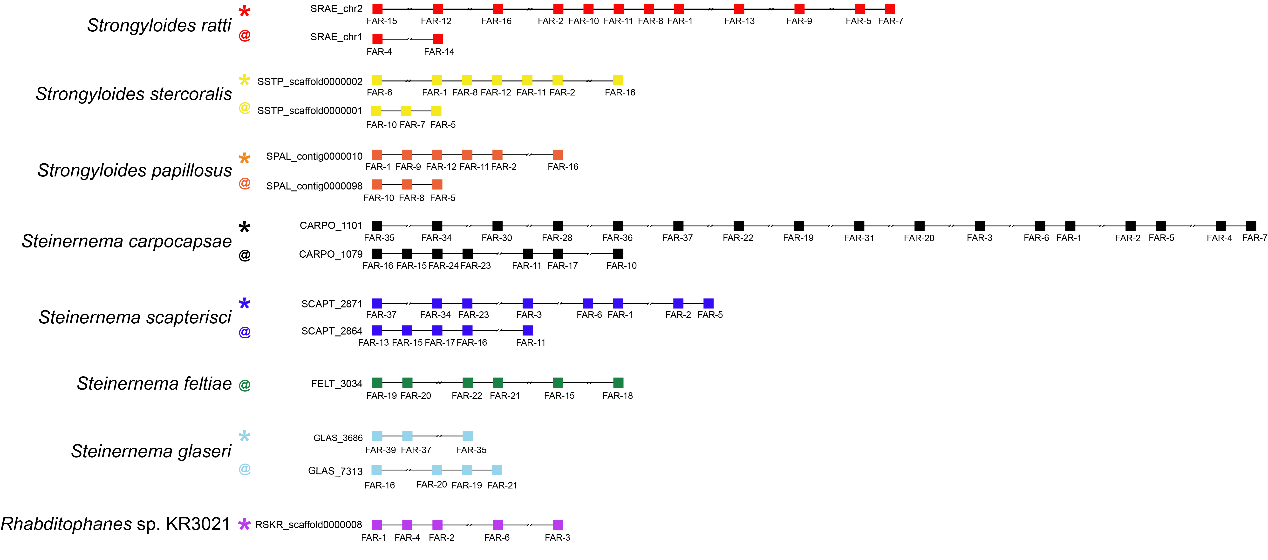


**Supplementary Figure S8** Tandem duplicated *far* gene in *Strongyloides ratti*, *Strongyloides stercoralis*, *Strongyloides papillosus*, *Steinernema carpocapsae*, *Steinernema scapterisci*, *Steinernema feltiae*, *Steinernema glaseri*, and *Rhabditophanes* sp. KR3021 from Clade IV. Square represents *far* gene on the scaffold or contig. Tandem genes were shown on the phylogenetic tree in Fig. 3.


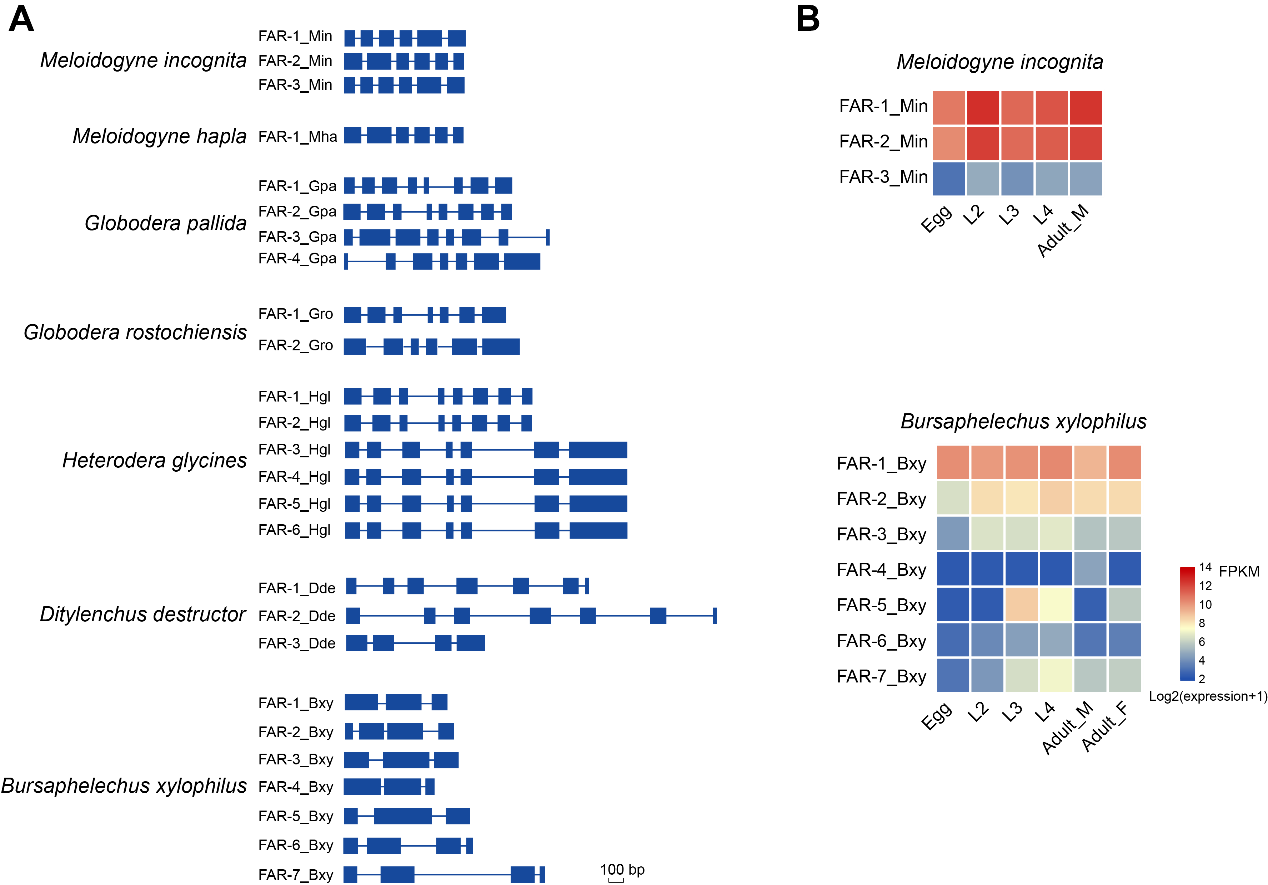


**Supplementary Figure S9** Gene structure and expression pattern of *far* in some plant-parasitic nematodes. (A) Intron analysis of *far* in some plant-parasitic nematodes. (B) Heatmap of the scaled gene expression of *far* genes in plant-parasitic nematodes. Expression of each gene was scaled between 2 (minimum expression - blue) and 14 (maximum expression - red) across developmental stages.


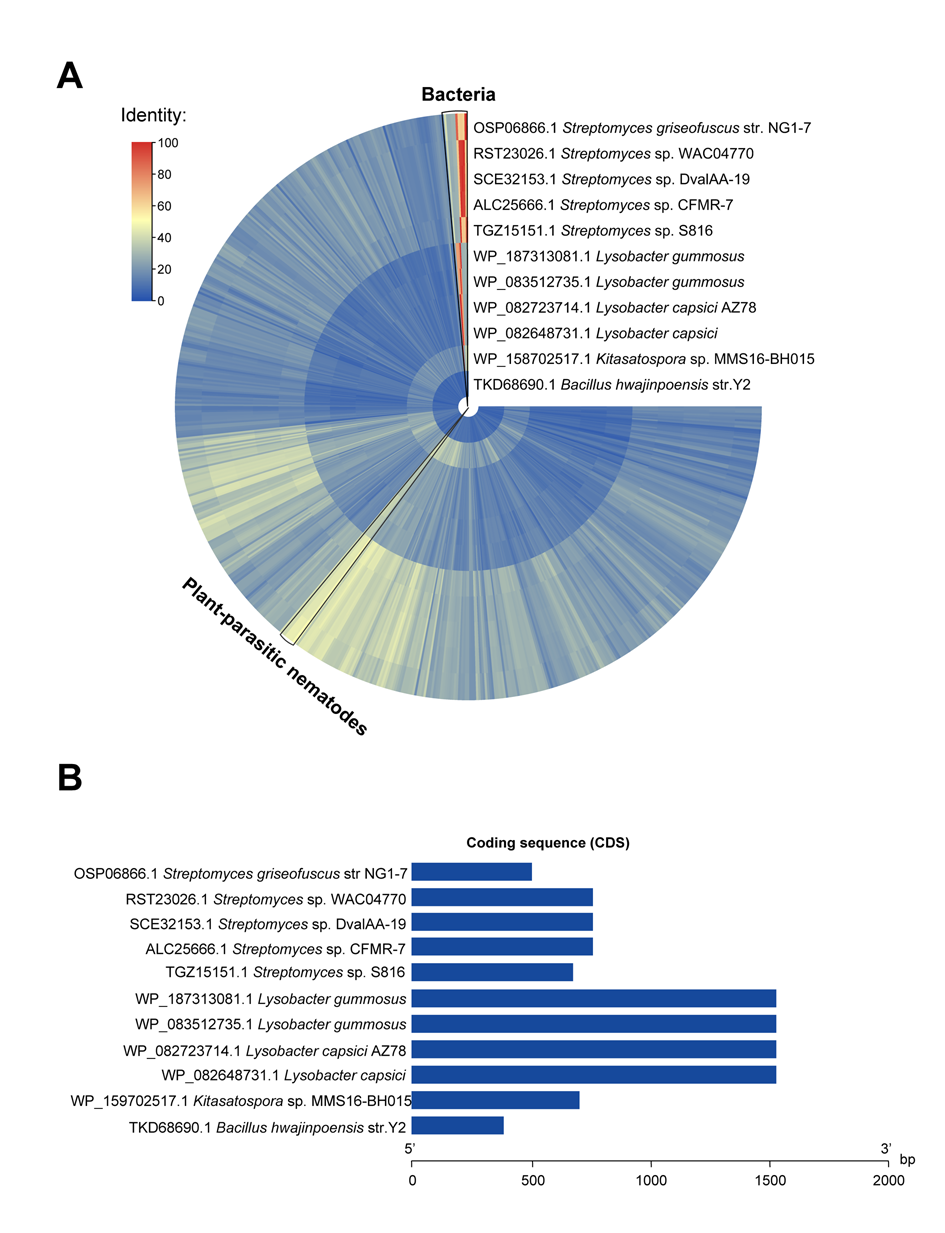


**Supplementary Figure S10** Sequence identity and gene structure analyses of bacteria *far*. (A) Heatmap shows the percentage of sequence identity of both FAR domains among nematodes and bacteria. (B) Gene structure of bacteria *far*.


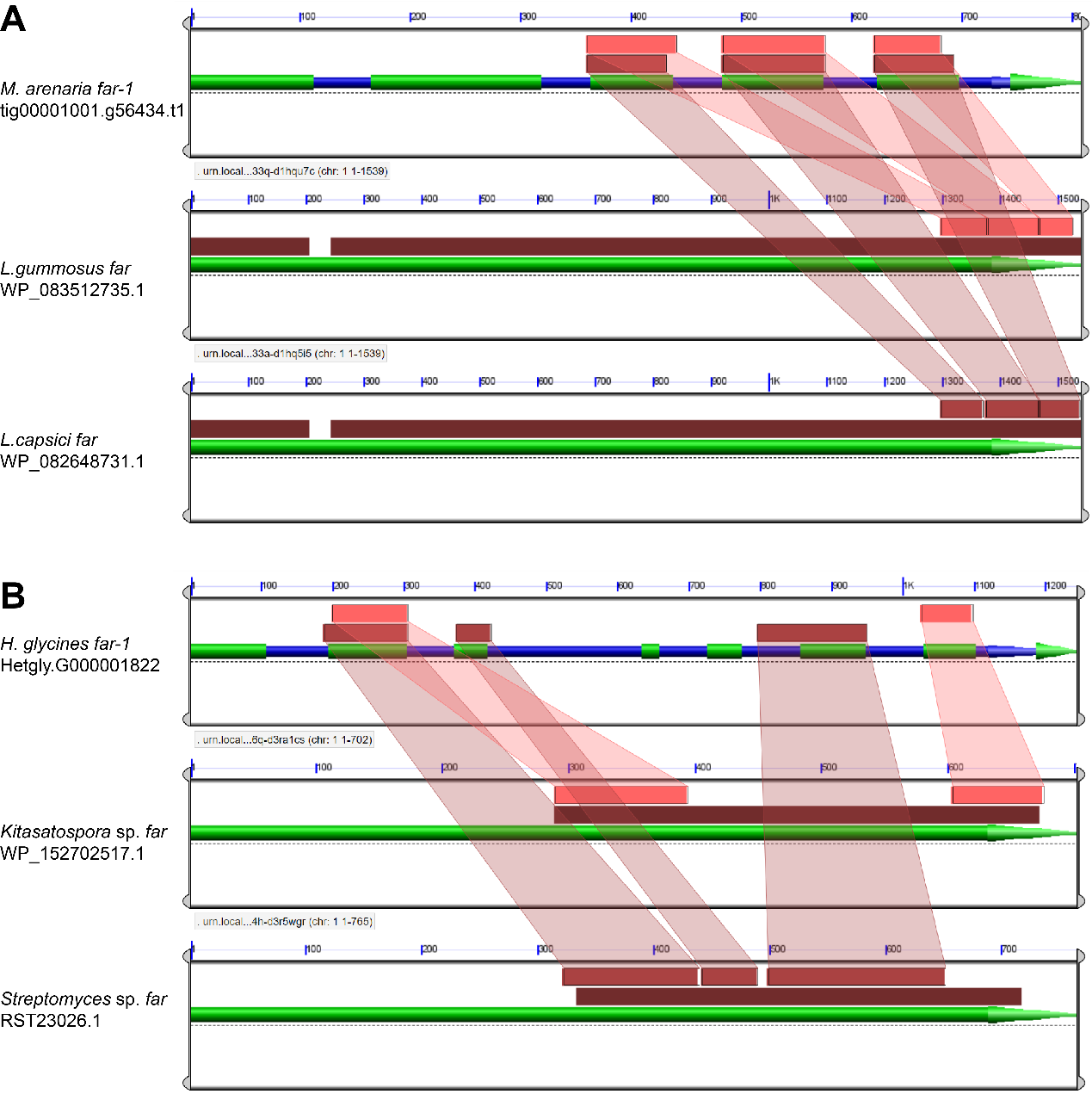


**Supplementary Figure** **S11** Genome colinearity in the CDS region of FAR domain between plant-parasitic nematodes and these bacteria. (A) Red shadow represents the synteny between plant parasitic nematode *Meloidogyne arenaria* and bacteria of two *Lysobacter* species. (B) Red shadow represents the synteny region between plant parasitic nematode *Heterodera glycines* and bacteria of *Kitasatospora* sp. and *Streptomyces* sp. Green cylinder represents exon; blue cylinder represents intron; red block represents syntenic region.


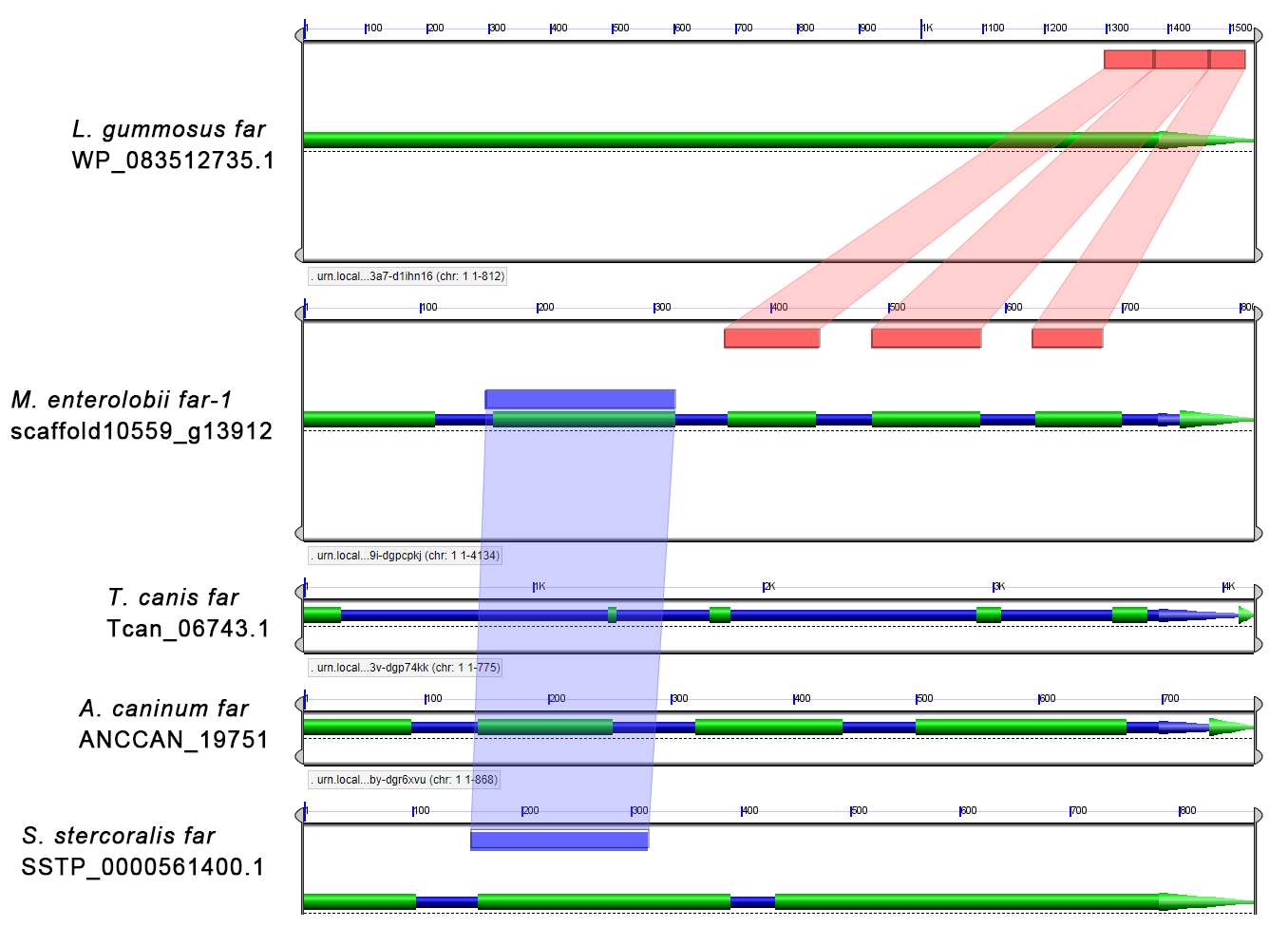


**Supplementary Figure S12** Genome colinearity in the CDS region of FAR domain among plant-parasitic nematodes, other nematodes from different clades, and these bacteria. Red shadow represents the synteny between plant parasitic nematode *Meloidogyne enterolobii* and bacteria of *Lysobacter gummosus*. Blue shadow represents the synteny between plant parasitic nematode *Meloidogyne enterolobii* and *Strongyloides stercoralis* (other nematodes). Plant parasitic nematode *Meloidogyne enterolobii* had no synteny with other nematodes of *Toxocara canis* and *Ancylostoma caninum.* Green cylinder represents exon; blue cylinder represents intron; red or blue block represent syntenic region.

**
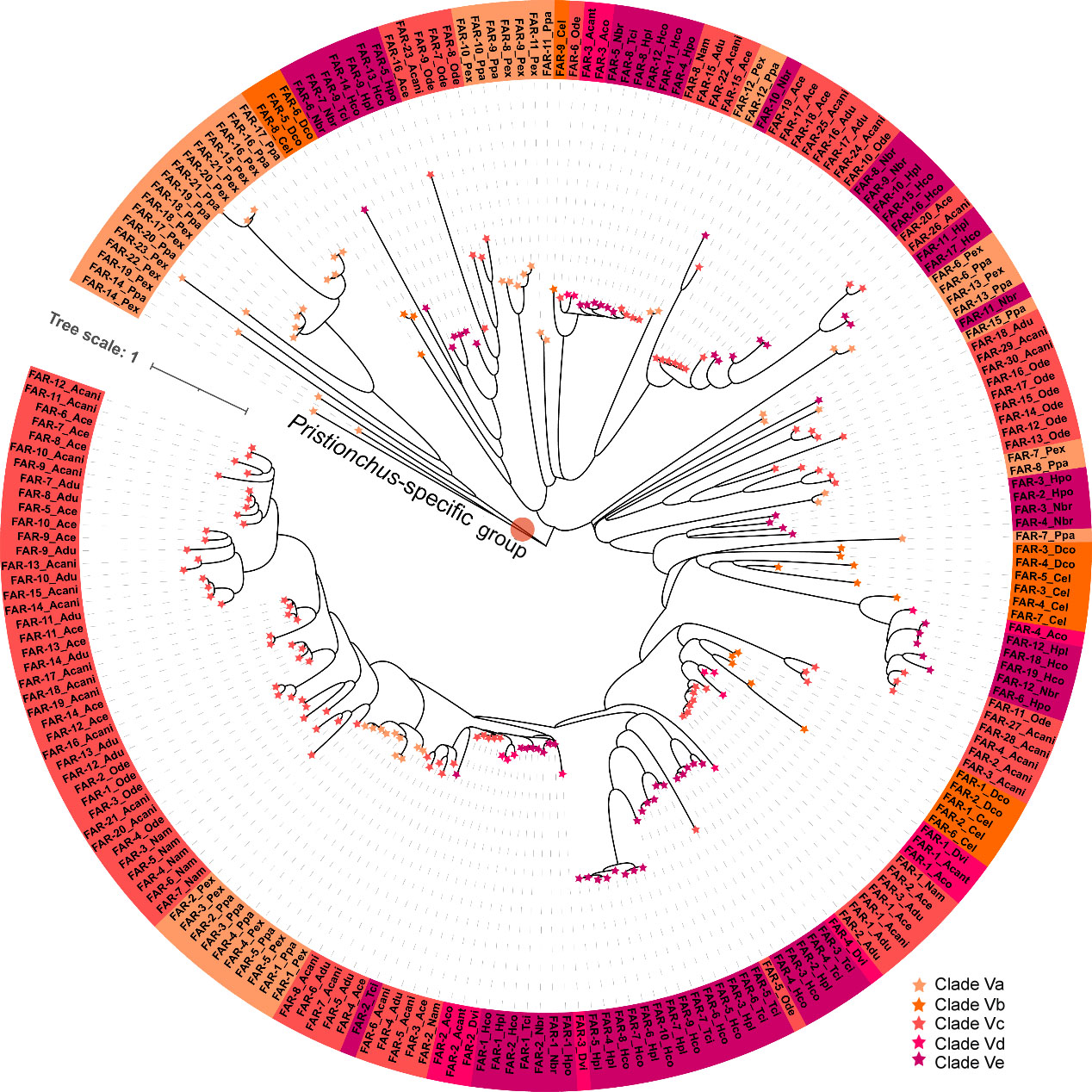
**

**Supplementary Figure S13** Protein Maximum Likelihood tree of FARs from nematodes in Clade V. Total 221 FAR proteins from nematodes in Clade V were divided into different groups in phylogenetic tree. Bootstrap values are shown in the nodes. The scale bar represents the number of amino acid substitutions per site. The red circle shows *Pristionchus*-specific group. The background color of gene name on the ring is corresponding to the color of star on the branch. The star with different colors indicates different subclades. Gene name includes the abbreviation of species name, as depicted in Fig.1.


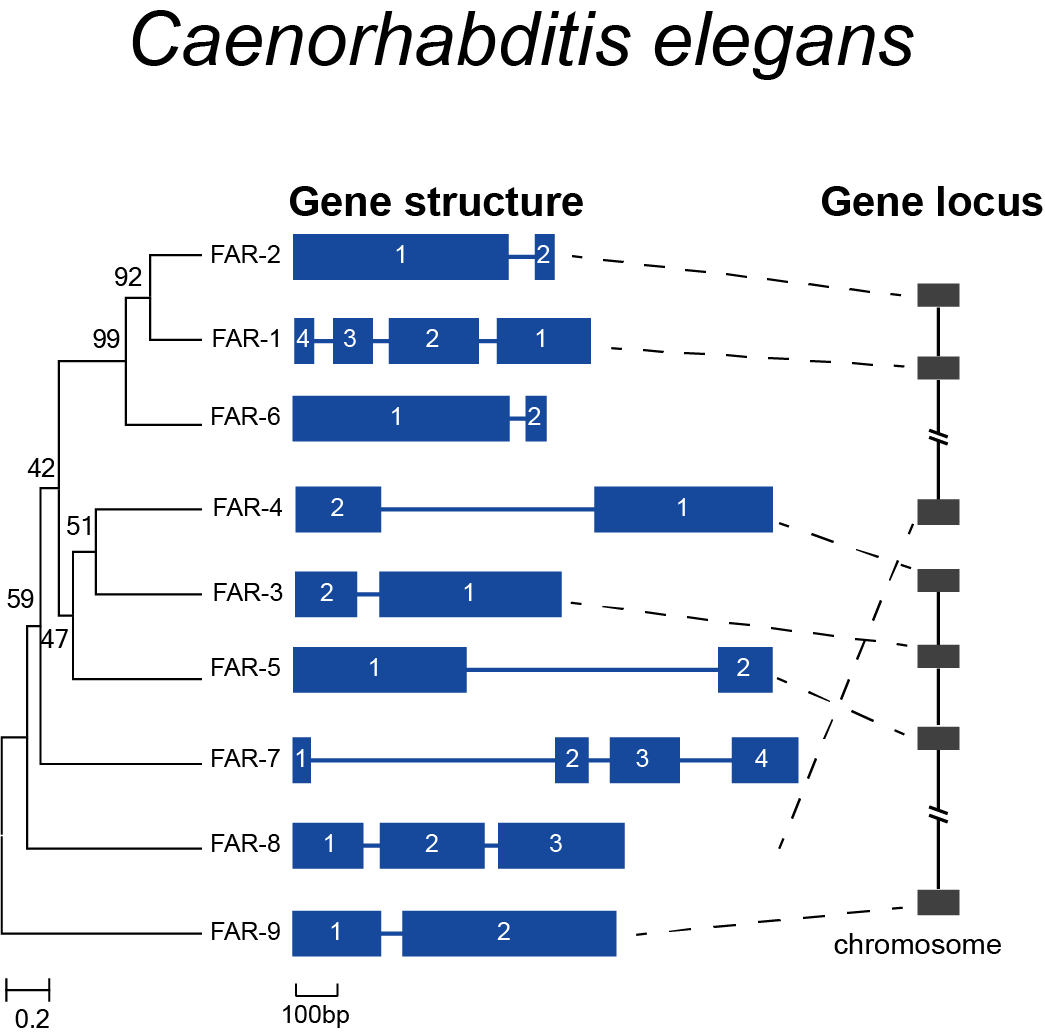


**Supplementary Figure S14** Intron analysis of tandem duplicated *C. elegans far.* Grey block represents tandem *far* gene on the chromosome. Blue rectangle represents exon; blue solid line represents intron; dotted line represents gene locus of tandem gene. Number in exon represents the order of exon in the gene.


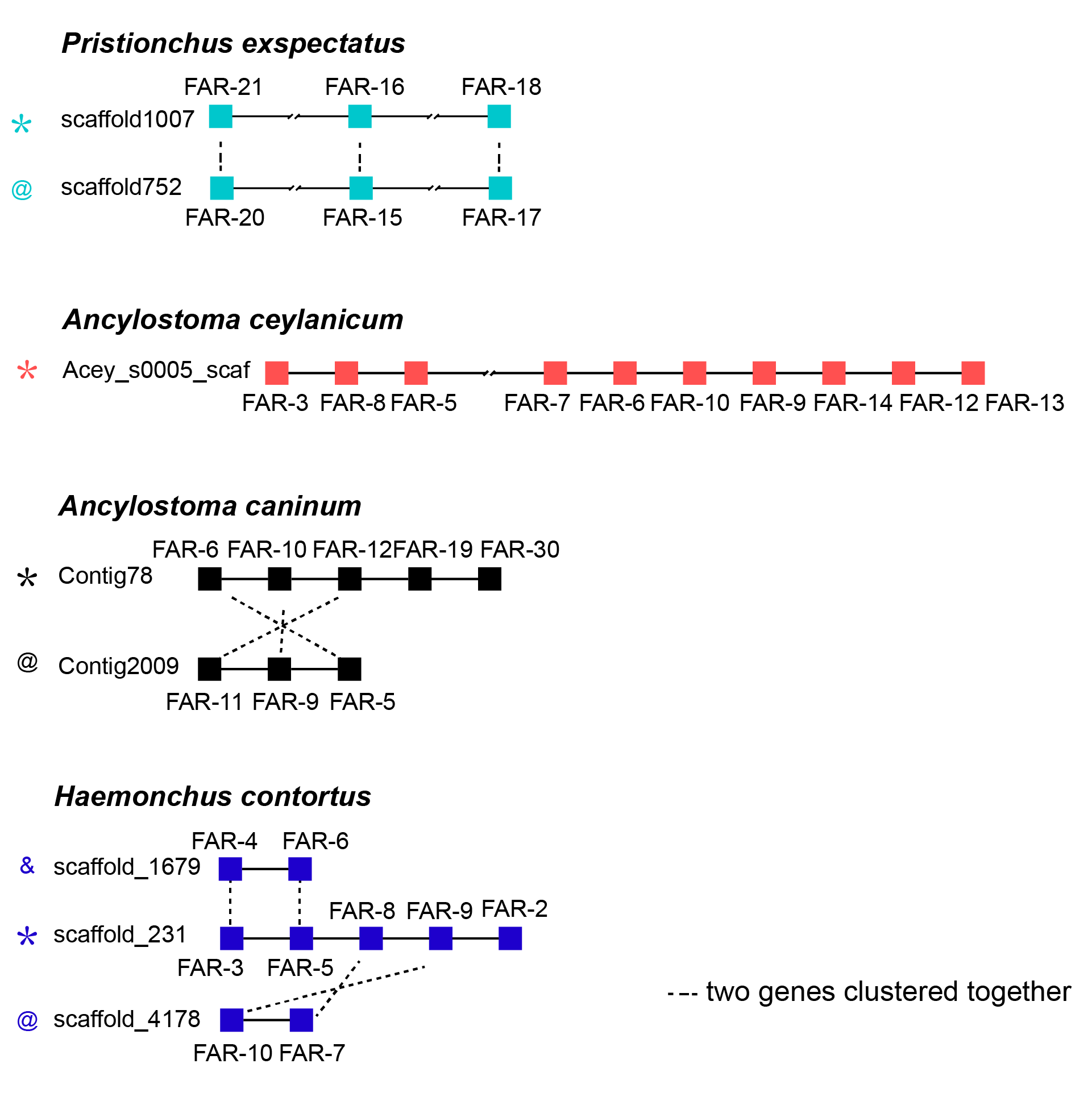


**Supplementary Figure S15** Gene locus of tandem duplicated *far* gene in *Pristionchus exspectatus*, *Ancylostoma ceylanicum*, *Ancylostoma caninum*, and *Haemonchus contortus* from Clade V. Square represents *far* gene on the scaffold or contig. Dotted line represents two genes clustered together on the phylogenetic tree in Fig. 5.


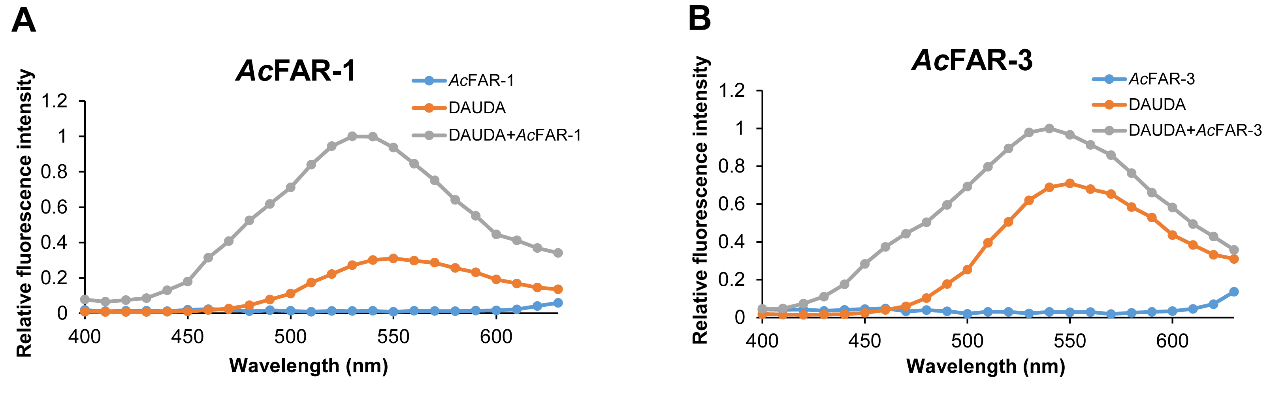


**Supplementary Figure S16** Relative fluorescence intensity of *Ac*FAR-1 and *Ac*FAR-3 binding with DAUDA.
