# Supplementary material for: Genus-level evolutionary relationships of FAR proteins reflect the diversity of lifestyles of free-living and parasitic nematodes
Source: BMC Biol. 2021 Aug 30;19:178. doi: 10.1186/s12915-021-01111-3 (PMC8407040; doi:10.1186/s12915-021-01111-3)
Supplement: Supplementary file 3 — Additional file 3: Table S2. Comparison of gene and exon numbers of far in nematodes. [file 12915_2021_1111_MOESM3_ESM.docx]

**Supplementary Table**

**Supplementary Table S2 Comparison of gene and exon numbers of *far* in nematodes**

| **Subclade** | **Species** | **Domain Number**  **(Gp-FAR-1>=100)** | **Gene Number** | **Exon Number** |
| --- | --- | --- | --- | --- |
| I | *Romanomermis culicivorax* | 0 | 0 | 0 |
|  | *Trichinella spiralis* | 0 | 0 | 0 |
|  | *Trichuris muris* | 0 | 0 | 0 |
|  | *Trichuris trichiura* | 0 | 0 | 0 |
|  | *Trichuris suis* | 0 | 0 | 0 |
| IIIa | *Enterobius vermicularis* | 5 | 5 | 5 |
|  | *Syphacia muris* | 2 | 2 | 4 |
| IIIb | *Anisakis simplex* | 2 | 2 | 4 |
|  | *Toxocara canis* | 3 | 3 | 5 |
|  | *Ascaris suum* | 4 | 4 | 5 |
|  | *Ascaris lumbricoides* | 5 | 5 | 6 |
| IIIc | *Dracunculus medinensis* | 3 | 3 | 4 |
|  | *Thelazia callipaeda* | 3 | 3 | 4 |
|  | *Dirofilaria immitis* | 3 | 3 | 5 |
|  | *Onchocerca flexuosa* | 1 | 1 | 3 |
|  | *Onchocerca ochengi* | 2 | 2 | 5 |
|  | *Onchocerca volvulus* | 3 | 3 | 6 |
|  | *Elaeophora elaphi* | 3 | 3 | 5 |
|  | *Acanthocheilonema viteae* | 3 | 3 | 4 |
|  | *Litomosoides sigmodontis* | 3 | 3 | 5 |
|  | *Loa loa* | 3 | 3 | 5 |
|  | *Wuchereria bancrofti* | 1 | 1 | 4 |
|  | *Brugia malayi* | 3 | 3 | 5 |
|  | *Brugia pahangi* | 3 | 3 | 5 |
| IVa | *Steinernema glaseri* | 40 | 40 | 4 |
|  | *Steinernema carpocapsae* | 37 | 37 | 3 |
|  | *Steinernema scapterisci* | 40 | 40 | 3 |
|  | *Steinernema feltiae* | 39 | 39 | 3 |
|  | *Steinernema monticolum* | 43 | 43 | 3 |
| IVb | *Rhabditophanes* sp. KR3021 | 8 | 8 | 3 |
|  | *Parastrongyloides trichosuri* | 19 | 19 | 2 |
|  | *Strongyloides stercoralis* | 16 | 16 | 2 |
|  | *Strongyloides ratti* | 16 | 16 | 2 |
|  | *Strongyloides venezuelensis* | 16 | 16 | 2 |
|  | *Strongyloides papillosus* | 16 | 16 | 2 |
| IVc | *Bursaphelenchus xylophilus* | 7 | 7 | 3 |
|  | *Ditylenchus destructor* | 3 | 3 | 6 |
|  | *Meloidogyne incognita* | 3 | 3 | 6 |
|  | *Meloidogyne hapla* | 1 | 1 | 6 |
|  | *Globodera pallida* | 4 | 4 | 8 |
|  | *Globodera rostochiensis* | 2 | 2 | 7 |
| Va | *Pristionchus exspectatus* | 23 | 23 | 7 |
|  | *Pristionchus pacificus* | 21 | 21 | 7 |
| Vb | *Diploscapter coronatus* | 6 | 6 | 6 |
|  | *Caenorhabditis elegans* | 9 | 9 | 3 |
| Vc | *Oesophagostomum dentatum* | 17 | 17 | 5 |
|  | *Necator americanus* | 8 | 8 | 5 |
|  | *Ancylostoma ceylanicum* | 20 | 20 | 5 |
|  | *Ancylostoma caninum* | 30 | 30 | 5 |
|  | *Ancylostoma duodenale* | 18 | 18 | 5 |
| Vd | *Dictyocaulus viviparus* | 4 | 4 | 5 |
|  | *Angiostrongylus cantonensis* | 3 | 3 | 6 |
|  | *Angiostrongylus costaricensis* | 4 | 4 | 7 |
| Ve | *Heligmosomoides polygyrus* | 6 | 6 | 6 |
|  | *Nippostrongylus brasiliensis* | 12 | 12 | 5 |
|  | *Teladorsagia circumcincta* | 9 | 9 | 5 |
|  | *Haemonchus contortus* | 19 | 19 | 6 |
|  | *Haemonchus placei* | 12 | 12 | 5 |
|  | **Total** | **586** | **586** | **247** |
