# Supplementary material for: Genus-level evolutionary relationships of FAR proteins reflect the diversity of lifestyles of free-living and parasitic nematodes
Source: BMC Biol. 2021 Aug 30;19:178. doi: 10.1186/s12915-021-01111-3 (PMC8407040; doi:10.1186/s12915-021-01111-3)
Supplement: Supplementary file 6 — Additional file 6: Table S6. Ligand binding ability of nematode FARs. [file 12915_2021_1111_MOESM6_ESM.docx]

**Supplementary Table**

**Supplementary Table S6 Ligand binding ability of nematode FARs**

| **Species** | **FAR** | **C14:0** | **C16:0** | **C18:0** | **C18:1** | **C18:4** | **Retinol** | **Reference** |
| --- | --- | --- | --- | --- | --- | --- | --- | --- |
| *Caenorhabditis elegans* | FAR-1 | - | - | - | - | √ | √ | [41] |
| *Necator americanus* | FAR-1 | √ | √ | √ | √ | √ | √ | [40] |
| *Ancylostoma ceylanicum* | FAR-1 | √ | √ | √ | √ | √ | √ | [43] |
| *Ancylostoma caninum* | FAR-1 | - | - | - | √ | - | √ | [21] |
| *Heligmosomoides polygyrus* | FAR-1 | - | - | - | √ | - | √ | [44] |
| *Angiostrongylus cantonensis* | FAR-1 | √ | √ | √ | √ | √ | √ |  |
| *Caenorhabditis elegans* | FAR-7 | - | - | - | ╳ | ╳ | √ | [42] |
| *Angiostrongylus cantonensis* | FAR-3 | ╳ | ╳ | ╳ | ╳ | ╳ | √ |  |

Note: √: strong binding ability with FAR; ╳: weak binding ability with FAR; -：non-detected.
